# Emerging Biomarkers and Nanobiosensing Strategies in Diabetes

**DOI:** 10.3390/bios15100639

**Published:** 2025-09-25

**Authors:** Anupriya Baranwal, Vipul Bansal, Ravi Shukla

**Affiliations:** 1 Sir Ian Potter NanoBioSensing Facility, NanoBiotechnology Research Laboratory, School of Science, RMIT University, Melbourne, VIC 3000, Australia; anupriyab3@gmail.com; 2 Centre for Advanced Materials & Industrial Chemistry, RMIT University, Melbourne, VIC 3000, Australia

**Keywords:** biosensor, diabetes, nanobiosensor, emerging biomarkers, microRNA, adipokine, type 1 diabetes, type 2 diabetes

## Abstract

Diabetes mellitus is a chronic metabolic disorder characterised by impaired glucose regulation, leading to severe complications affecting multiple organ systems. Current diagnostic approaches primarily rely on glucose monitoring, which, while being effective, fails to capture the underlying molecular changes associated with disease progression. Emerging biomarkers such as microRNAs (miRNAs) and adipokines offer new insights into diabetes pathophysiology, providing potential diagnostic and prognostic value beyond traditional methods. Given this, precise monitoring of the altered levels of miRNAs and adipokines can forge a path towards early diabetes diagnosis and improved disease management. Biosensors have revolutionised diabetes diagnostics, with glucose biosensors dominating the market for decades. However, recent advancements in nanobiosensors have expanded their scope beyond glucose detection, enabling highly sensitive and selective monitoring of biomolecular markers like miRNAs and adipokines. These nanotechnology-driven platforms offer rapid, inexpensive, and minimally invasive detection strategies, paving the way for improved disease management. This review provides an overview of diabetes, along with its pathogenesis, complications, and demographics, and explores the clinical relevance of miRNAs and adipokines as emerging biomarkers. It further examines the evolution of biosensor technologies, highlights recent developments in nanobiosensors for biomarker detection, and critically analyses the challenges and future directions in this growing field.

## 1. Diabetes Mellitus: An Overview

Diabetes mellitus is a group of chronic metabolic disorders characterised by elevated blood glucose levels, commonly known as hyperglycaemia [1]. This condition arises due to defects in insulin secretion, insulin action, or both [1,2]. Insulin is a peptide hormone secreted by the pancreatic β-cells. It plays a pivotal role in regulating blood glucose by facilitating glucose uptake into cells for energy production or storage [3]. Moreover, insulin also regulates carbohydrate, lipid, and protein metabolism while promoting cell division by demonstrating mitogenic effects. According to the World Health Organisation (WHO), diabetes is a major global health issue, with an alarming increase in prevalence, particularly in low- and middle-income countries [4]. Diabetes is broadly classified into several types, with type 1 diabetes (T1D) and type 2 diabetes (T2D) being the most common forms [1,2,4].

### 1.1. Type 1 Diabetes (T1D)

**Figure 1:**
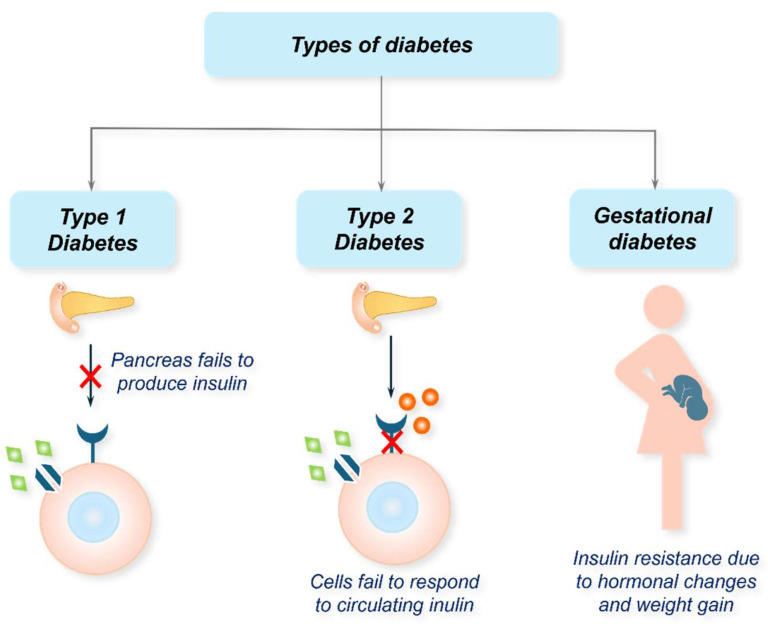
Schematic illustration of types of diabetes. Among the three types, type 1, type 2 and gestational diabetes, type 2 is most common and contributes to an estimate of 90–95% of all diabetes cases.

T1D, often referred to as juvenile diabetes, is an autoimmune condition where the body’s immune system destroys insulin-producing pancreatic β-cells (Figure 1). This leads to an absolute insulin deficiency, requiring individuals to depend on exogenous insulin for survival [1,2]. The exact aetiology behind T1D remains unknown, but genetic predisposition and environmental triggers, such as viral infections, are believed to play a role [5]. Considering the multi-factor requirement to induce T1D would mean that the condition is uncommon; however, it is not rare. T1D accounts for approximately 5–10% of all diabetes cases and commonly manifests in childhood or adolescence, although it can develop at any age [1,5]. In 2021, approximately 8.4 million individuals across the globe had TID, and about 510,000 new cases were reportedly diagnosed in that year alone [6]. Of this estimate, 18% were younger than 20 years, 64% were aged between 20 and 59 years, and the remaining were 60 years or older. About 35,000 undiagnosed individuals died within one year of symptom onset. The mortality rates between countries vary greatly depending on their economic status, as 20% of individuals with T1D were in low-income and lower-middle-income countries [6].

T1D is known to develop in a clinically silent manner over a period of a few months to years. The disease progression can be divided into three stages once the commencement of autoimmunity has occurred [7,8]. Stage 1 is marked by the presence of two or more autoantibodies, stage 2 encompasses autoantibodies from stage 1 and β-cell dysfunction due to progressive loss of β-cell mass, and the clinical manifestation of T1D defines stage 3. Notably, individuals diagnosed in stage 1 exhibit a risk of 35–50% in progressing to stage 3 of T1D within 5–6 years. The risk rises to 75% for individuals being diagnosed in stage 2, with clinical manifestation of T1D in 2 years [8,9].

### 1.2. Type 2 Diabetes (T2D)

**Figure 2:**
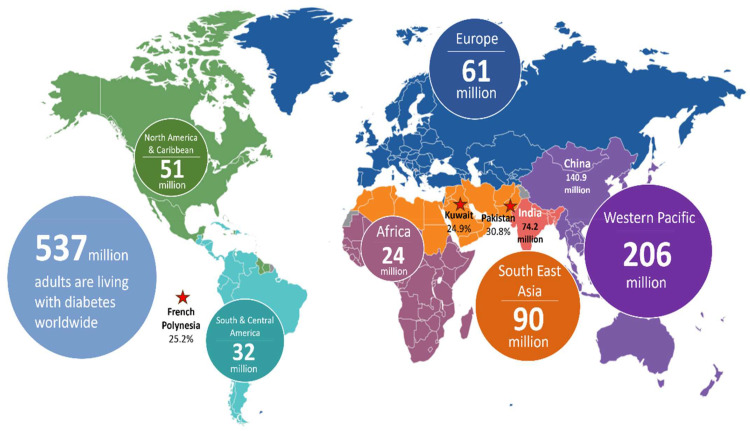
Global diabetes demographics in 2021. Data is sourced from the IDF Diabetes Atlas 2021 [12].

T2D is a complex multi-factor polygenetic disease that is primarily driven by preobesity/obesity [10]. It is characterised by insulin resistance, where the insulin-sensitive tissues fail to respond effectively to insulin (Figure 1), coupled with a progressive decline in pancreatic insulin production [11]. Consequently, elevated levels of blood glucose (hyperglycaemia) become evident in individuals with the condition. T2D primarily affects adults but is increasingly being diagnosed in younger populations due to rising obesity rates, an ageing population, and sedentary lifestyles [10]. According to the International Diabetes Federation (IDF), ~537 million adults aged 20–79 years are living with diabetes across the globe (Figure 2) [12]. If the current trends continue, this figure is expected to rise to 783 million by 2045 [13]. Of this estimate, T2D accounts for about 90–95% of cases. In 2021, high body-mass-index (BMI) was reported to contribute over 50% of the global T2D disability-adjusted life year (DALY). Accumulating reports indicate a correlation between T2D and gender difference, with males showing a higher prevalence than females [14]. Age is another important risk factor for T2D, with a rise of 24.4% in diabetes in the 75–79 years age group; however, a >1% increment was evident for the under 20 age group [15]. Other factors contributing to the prevalence of T2D include poor dietary choices, usage of tobacco, alcohol consumption, environmental factors, and physical inactivity [15]. Aside from this, genetics plays a small yet crucial role in diabetes, with an estimated heritability of 30% to 70%, depending on the age of diabetes onset and the glycaemic status of diabetic cases [16].

Globally, diabetes accounts for 12% of all health expenditures, amounting to over $966 billion USD annually [15]. This includes costs for medication, hospitalisations, and management of complications. Beyond financial costs, diabetes imposes a significant quality-of-life burden due to its chronic nature and associated complications. Efforts to mitigate the disease burden are multi-faceted, including early detection, lifestyle interventions, and advanced treatments [10]; however, healthcare disparities and the complications of diabetes management continue to pose their own challenges.

### 1.3. Other Types

In addition to T1D and T2D, there are other forms of diabetes, such as gestational diabetes mellitus (GDM) and monogenic diabetes [17,18]. GDM occurs during pregnancy when hormonal changes lead to insulin resistance (Figure 1), potentially affecting both maternal and foetal health [17]. While GDM usually resolves after childbirth, it increases the risk of developing T2D later in life. Monogenic diabetes, a rare form, is caused by mutations in a single gene affecting insulin production or function [18]. It includes maturity-onset diabetes of the young and neonatal diabetes. Another noteworthy type is secondary diabetes, which arises due to underlying conditions like pancreatic diseases, hormonal disorders, or medication side effects.

### 1.4. Secondary Complications

**Figure 3:**
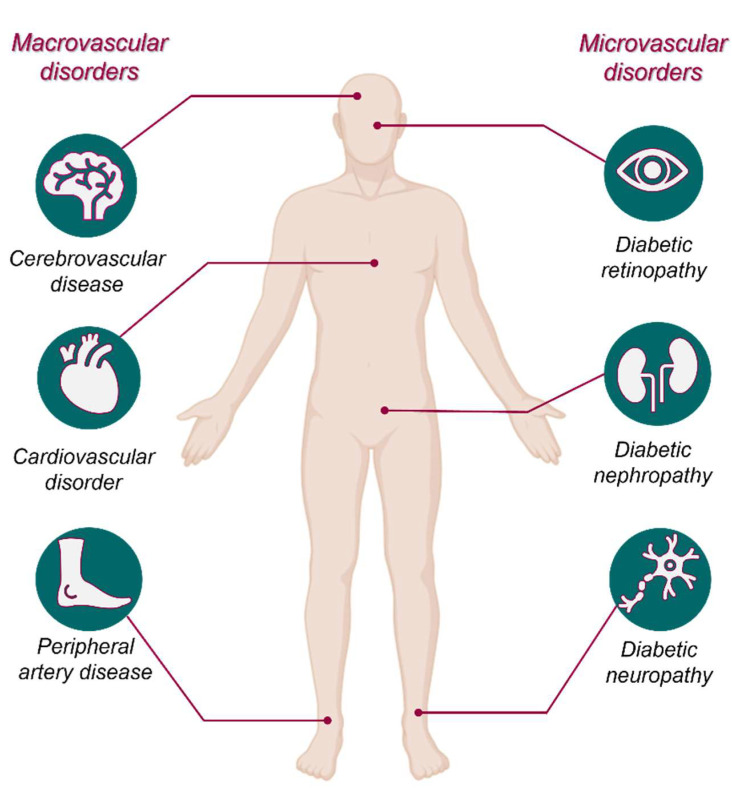
Microvascular and macrovascular complications associated with diabetes.

Prolonged hyperglycaemia can lead to several debilitating complications affecting various organ systems (Figure 3). Microvascular complications include diabetic retinopathy, which can cause vision loss, diabetic nephropathy leading to chronic kidney disease, and diabetic neuropathy, resulting in numbness, pain, or ulcers [19]. Macrovascular complications, such as cardiovascular disease, stroke, and peripheral artery disease, are common in diabetes patients and are significant contributors to morbidity and mortality [19]. Poorly managed diabetes also impairs immune function, increasing susceptibility to infections like urinary tract infections and skin disorders. Additionally, diabetic foot ulcers, often exacerbated by neuropathy and poor circulation, can lead to severe outcomes, including amputation if untreated [20]. Preventing complications requires a comprehensive approach, including regular health check-ups, strict glycaemic control, and managing comorbidities like hypertension and dyslipidaemia.

### 1.5. Diabetes Symptoms

The symptoms of diabetes vary depending on the type and severity of the condition. Common symptoms include polyuria (frequent urination), polydipsia (increased thirst), and polyphagia (excessive hunger) [1,4]. Unexplained weight loss, fatigue, excessive skin dryness, and blurred vision are also hallmark signs of the disease [4]. Additional signs of poorly managed diabetes include slow-healing wounds, recurrent infections, and numbness or tingling in the hands and feet due to nerve damage [4]. In GDM, symptoms are typically mild and may overlap with normal pregnancy-related changes, making routine screening critical for diagnosis.

## 2. Diabetes Screening and Diagnosis

Diabetes is a chronic condition characterised by persistent hyperglycaemia, which necessitates early and accurate diagnosis to facilitate timely interventions, effective disease management, and the prevention of secondary complications. The approaches of screening and diagnosis vary between T1D and T2D due to their distinct pathophysiological mechanisms and clinical presentations. For both types, biomarkers play a pivotal role in mediating early detection and risk stratification. Biomarkers, as defined by the National Institute of Health (NIH), are detectable indicators of biological processes, pathogenic conditions, or responses to interventions, and they include molecular, cellular, and physiological characteristics [21]. Their utility spans across several categories, including diagnostic, predictive, prognostic, monitoring, response, and safety biomarkers [21,22]. For the purpose of this review, however, the primary focus will be on traditional and potential diagnostic and predictive biomarkers.

### 2.1. T1D Screening and Diagnosis

Screening for T1D focuses on identifying individuals at risk of developing the disease before the onset of clinical symptoms. Unlike T2D, which has a gradual onset [10], T1D often presents acutely [2]. Screening efforts are typically targeted at individuals with a family history of T1D or other autoimmune disorders. The primary tools for screening include the detection of islet autoantibodies, such as glutamic acid decarboxylase antibodies (GADA), insulin autoantibodies (IAA), islet antigen-2 antibodies (IA–2A), and zinc transporter 8 antibodies (ZnT8A) [23,24,25]. These autoantibodies typically appear several months or years prior to the clinical onset and serve as predictive indicators of β-cell destruction. The presence of two or more autoantibodies in an individual is associated with a high risk of developing T1D [26]. Human leukocyte antigen (HLA) class II genotyping is another means to identify genetic susceptibility to T1D. Reportedly, specific HLA haplotypes, such as HLA DR-DQ, confer a significantly increased risk of developing T1D [27,28]. However, genetic screening is less commonly performed due to the variability in genetic predisposition and its limited predictive value. Screening is particularly relevant in research and preventive settings, as it allows for the identification of susceptible individuals who might benefit from immunotherapies or lifestyle interventions to delay or prevent disease onset. Despite these advances, routine population-wide screening for T1D has not yet been adopted due to the lack of established, widely effective preventive measures and the often sudden onset of clinical symptoms [29].

The diagnosis of T1D typically occurs when individuals exhibit symptoms of hyperglycaemia, such as polydipsia, polyuria, unintended weight loss, fatigue, and, in severe cases, diabetic ketoacidosis (DKA) [30]. DKA is a life-threatening condition characterised by the accumulation of ketone bodies in the blood, which leads to metabolic acidosis. This condition manifests as nausea, vomiting, abdominal pain, rapid breathing, and confusion [31]. Research shows that over 95% of newly diagnosed patients seek medical attention due to the presence of the aforementioned symptoms [32], while only a small percentage are diagnosed through routine glucose tests or the detection of autoantibodies as part of longitudinal screening programmes [7].

The diagnostic criteria for T1D are based on blood parameters like glucose and HbA1c levels, which reflect the glycaemic status in an individual. A fasting blood glucose (FBG) level of ≥126 mg/dL (≥7.0 mmol/L) indicates hyperglycaemia after at least 8 h without food, while a random blood glucose (RBG) level of ≥200 mg/dL (≥11.1 mmol/L) in the presence of symptoms confirms diabetes [33]. The oral glucose tolerance test (OGTT) is a strategy in which plasma glucose is measured 2 h after ingesting a glucose solution (2 h postprandial). OGTT result is diagnostic if the level reaches ≥200 mg/dL (≥11.1 mmol/L) [33], reflecting impaired glucose regulation. An HbA1c level of ≥6.5% indicates chronically elevated blood sugar levels over the previous 2–3 months but may be less specific in T1D due to its rapid onset [34]. To confirm T1D, additional tests such as C-peptide levels and autoantibody testing are often performed. Low or undetectable C-peptide levels, which measure endogenous insulin production, are indicative of β-cell failure characteristic of T1D [35,36]. Autoantibody testing helps differentiate T1D from other forms of diabetes, particularly in atypical cases or when the clinical presentation is ambiguous [25]. Early and accurate diagnosis is essential to promptly initiate insulin therapy, prevent complications like DKA, and achieve optimal glycaemic control.

### 2.2. T2D Screening and Diagnosis

Screening for T2D is a proactive approach aimed at identifying individuals with undiagnosed diabetes or those at high risk of developing the disease. Unlike T1D, which typically presents acutely, T2D develops slowly and often remains asymptomatic in its early stages [37]. Screening is particularly important for individuals with risk factors such as preobesity/obesity, advanced age, a family history of diabetes, or a history of gestational diabetes [10]. Preobesity/obesity is defined by body mass index (BMI), with a BMI of ≥25 kg/m^2^ (≥23 kg/m^2^ in Asia) as preobese and ≥30 kg/m^2^ (≥27.5 kg/m^2^ in Asia) as obese [38]. However, BMI alone cannot be used as an alternative for screening total fat content in individuals, as people with the same BMI may have different fat content and distribution, representing different risks of diabetes [39]. Waist-to-height ratio, defined as waist circumference (cm) divided by height (cm), has been proposed as a substitute indicator of BMI to define obesity [40]. In addition to risk factors, commonly used approaches to screen diabetes include FBG and HbA1c tests. An HbA1c level between 5.7% and 6.5%, termed prediabetes [41], reflects intermediate levels of chronic hyperglycaemia and signifies an increased risk of progression to T2D. Similarly, an FBG level between 100 and 125 mg/dL (5.6–6.9 mmol/L) indicates impaired fasting glucose, another marker of elevated diabetes risk [42]. Screening is recommended for adults aged ≥ 35 years and younger individuals with significant risk factors [37]. Regular screening enables early identification of at-risk individuals, allowing for timely lifestyle interventions or pharmacological measures to delay or prevent the onset of diabetes and its complications.

The diagnosis of T2D relies on standardised criteria similar to those used for T1D; however, distinguishing between the two types is essential due to differences in their underlying pathophysiology and management strategies. Diagnostic tests include FBG, HbA1c, RBG with symptoms of hyperglycaemia, or OGTT results showing a 2 h postprandial plasma glucose (2 h PG). The diagnostic threshold set by the American Diabetes Association (ADA) and the WHO for each criterion is captured in Table 1. These thresholds apply to both T1D and T2D, as they reflect the presence of hyperglycaemia severe enough to warrant a diagnosis of diabetes. In T2D, symptoms such as polyuria, polydipsia, and fatigue may develop gradually or be absent entirely, especially in the early stages. Recently, the IDF recognised that individuals with 1 h postprandial glucose (1 h PG) 8.6 mmol/L would be considered prediabetic, while ≥11.6 mmol/L would be diabetic [43]. Unlike T1D, C-peptide levels are typically normal or elevated in T2D due to ongoing endogenous insulin production, albeit insufficient to overcome insulin resistance [36]. Often, T2D is diagnosed incidentally during routine medical checkups or evaluations for related conditions such as hypertension, dyslipidaemia (abnormal levels of lipids in the blood), or cardiovascular disease [10].

## 3. Emerging Biomarkers in Diabetes

The diagnostic criteria mentioned above have established their importance in routine clinical practice due to their ease of measurement, strong correlation with long-term diabetes, and ability to guide therapeutic decisions. However, they lack the sensitivity to identify diabetes in its early or preclinical stages. This makes it difficult to judge whether changes in these parameters lead to diabetes or the onset of diabetes changes these parameters and biomarkers. In view of this, biomarkers exhibiting higher specificity and sensitivity are emerging as potential solutions to facilitate early intervention and reduce the risk of diabetes-related complications.

### 3.1. Emerging Biomarkers in T1D

Over the years, improved technologies for monitoring and insulin administration have significantly reduced the disease burden and improved life quality; however, T1D remains incurable. Today, when T1D is clinically established, >80% of the total β-cell mass is lost and secondary complications start becoming evident [45,46]. To prevent or delay the onset of these complications, early identification of individuals at risk of developing T1D is required, ideally prior to or independent of hyperglycaemic condition. Therefore, novel biomarkers that can identify such individuals are being searched using candidate-driven and/or screening approaches.

In the last decade, circulating microRNAs (miRNAs) have emerged as potential biomarkers for various diseases as their level gets altered with different pathological conditions [47,48,49,50]. miRNAs are short (21–24 nucleotides long) non-protein coding RNAs that play regulatory roles in post-transcriptional modifications, gene expression, and pathophysiological processes like metabolism, inflammation, and immune response [50]. Several miRNAs have been identified for their physiological involvement in tissues affected with T1D and related complications (Table 2). Their aberrant expression profile in various biological fluids, including serum, plasma, and urine, makes them accessible and minimally invasive diagnostic biomarkers. The presence of miRNAs in circulation is either to mediate inter-cellular communication or due to stressed/ruptured cells [51]. While RNAs are generally unstable and prone to degradation by nucleases, miRNAs exhibit remarkable stability in body fluids due to their association with protective carriers such as exosomes, lipoproteins, and RNA-binding proteins. These associations shield miRNAs from enzymatic degradation, making them reliable biomarkers for diagnostic applications [50]. In addition, they are resistant to repeated freeze–thaw cycles, pH fluctuations, and high temperatures, exhibit a long half-life [52] and can be accurately measured [53] from stored clinical samples.

In recent years, several other novel biomarkers have been explored, quantified, and tested for their role in predicting T1D onset and progression and risk stratification of individuals with and/or without T1D. Details on these biomarkers can be found in reviews [54,55,56,57,58,59] as their discussion falls out of the scope of this review. 

**Table 2:** miRNA in diabetes and associated complications.

miRNA	Diseases
miR-145-5p	T1D [51], T2D [60,61,62], diabetic nephropathy [63], microvascular complications [64,65], macrovascular complications [66], diabetic foot ulcer [67,68], gestational diabetes [69]
miR-30e-3p	T1D [70], prediabetes [71], T2D [72], microvascular complication [73], macrovascular complication [74], retinopathy [75], diabetic nephropathy [76]
miR-30c-5p	T2D [77], diabetic macrovascular complication [78], diabetic nephropathy [79,80]
miR-148a-3p	T1D [70,81,82], T2D [83], diabetic retinopathy [75]
miR-155-5p	T1D [84,85], T2D [86], diabetic dyslipidaemia [87], diabetic foot ulcer [88]
miR-25-3p	T1D [82], diabetic nephropathy [89], diabetic retinopathy [90]
miR-625-5p	Diabetic cognitive impairment [91]
miR-20a-5p	Diabetic foot ulcer [92], gestational diabetes [93], diabetic macrovascular complication [94,95], non-alcoholic fatty liver disease [96,97]
miR-326	T1D [51,98], T2D [90]
miR-24-3p	T1D [51,81,82,99], T2D [83], gestational diabetes [100], diabetic retinopathy [101,102]
miR-301b-3p	T1D [51], diabetic wound healing [103]
miR-223-3p	T1D [51], T2D [104], diabetic microvascular complication [105], gestational diabetes [106]
miR-21-5p	T2D [83], diabetic nephropathy [89,107], diabetic foot ulcer [92]
miR-30a-5p	T1D [51,82], T2D [108] and prediabetes [109], diabetic nephropathy [76,110]
miR-186-5p	T1D [51], diabetic peripheral neuropathy [111], diabetic macrovascular complication [112,113], diabetic retinopathy [114]
miR-26a-5p	T1D [51,82], T2D [60,83], diabetic nephropathy [89]
miR-126-3p	T2D [60,83,115,116,117], diabetic retinopathy [118,119], diabetic nephropathy [76,89]
miR-199a-3p	T1D [51], T2D [60], diabetic macrovascular complication [78], diabetic retinopathy [75]
miR-127-3p	T1D [51,120], T2D and prediabetes [121]
let-7e-5p	T1D [51,99], T2D [83] and prediabetes [121], diabetic retinopathy [75], diabetic nephropathy [107]
miR-146a-5p	T1D [51,81], T2D [83,122,123] and prediabetes [124]
miR-200a-3p	T1D [51,82,125], diabetic retinopathy [126], diabetic nephropathy [127,128], diabetic macrovascular complication [129]
miR-34a-5p	T1D [51,130], T2D [122,124], diabetic cognitive impairment [131], diabetic cataractogenesis [132]
miR-181a-5p	T1D [51,81,82], T2D [133], diabetic nephropathy [134]
miR-22-5p	T1D [51], macrovascular complications [135]
miR-93-5p	T1D [70], diabetic macrovascular complication [136]

### 3.2. Emerging Biomarkers in T2D

The increasing global burden of T2D emphasises the urgent need for precise biomarkers that can predict disease onset, monitor its progression, and provide insights into prognosis. Recent advances in molecular biology and high-throughput technologies have facilitated the discovery of novel biomarkers with significant clinical utility [137]. These biomarkers can be categorised into genetic, molecular, metabolic, inflammatory, and microbiome-related factors (Table 3). Each of these factors capture distinct aspects of T2D pathophysiology, including insulin resistance, β-cell dysfunction, and chronic inflammation. Detailed information on emerging biomarkers in T2D prediction, diagnosis, and prognosis can be found in reviews [138,139,140,141,142,143,144].

While genetic, epigenetic, and metabolomic markers have significantly advanced the understanding of T2D pathophysiology [182], adipokines offer a distinct advantage as biomarkers due to their direct contribution to the metabolic and inflammatory pathways underpinning the disease [183]. Unlike genetic and epigenetic markers, which often indicate predisposition, adipokines reflect the real-time disease status by revealing the functional state of adipose tissues. Adipokines or adipocytokines are bioactive proteins and peptides secreted by the adipose tissues. They are known to function as endocrine and paracrine factors to regulate energy homeostasis, glucose and lipid metabolism, insulin sensitivity, inflammation, and immune response [184]. The secretion patterns of adipokines are dynamically modulated by metabolic and environmental factors, which makes them highly sensitive to the systemic changes occurring in T2D [183]. Moreover, they bridge the gap between obesity and T2D by providing mechanistic insights into how adipose tissue dysfunction promotes insulin resistance and chronic inflammation [183]. Various adipokines have been implicated in T2D pathogenesis, with some exhibiting synergistic effects while others imparting antagonistic effects (Table 4). For instance, in the early stages of metabolic dysfunction, anti-inflammatory adipokines like adiponectin and vaspin may counterbalance the deleterious effects of pro-inflammatory adipokines like leptin and resistin [185]. Owing to these attributes, adipokines are emerging as not only predictive but also actionable biomarkers with potential applications in both diagnosis and personalised therapeutic strategies.

Like T1D, various circulating miRNAs have also been associated with T2D (Table 2). T2D pathogenesis is silent in nature, which develops slowly over several years and often goes unnoticed due to its asymptomatic nature. Traditional biomarkers, despite being the gold standard in T2D diagnosis, only monitor hyperglycaemia—a condition developed in the later stage of T2D pathogenesis, indicating their inadequacy in T2D prediction. In view of this, miRNAs are emerging as excellent alternatives. Reportedly, miRNA expression profiles become altered in body fluids several years prior to the clinical manifestation of T2D [50,142,203]. Therefore, accurate detection of circulating miRNAs in body fluids can help in mediating early T2D diagnosis. While this seems ideal, precise monitoring of miRNA levels is challenging due to their small size (21–24 nucleotides), sequence homology, and low abundance in biological fluids.

Over the years, a wide range of biomolecular assays have been developed and applied for the detection of miRNAs, including microarray technology [204], quantitative reverse transcription polymerase chain reaction (qRT-PCR) [205,206], rolling circle amplification (RCA) [207,208], and ligase chain reaction (LCR) [209]. While these techniques have advanced miRNA detection by offering high sensitivity and specificity, they are often constrained by inherent limitations. Microarray technology, for instance, requires labour-intensive hybridisation steps and specialised equipment [210]. Similarly, qRT-PCR, a gold standard for nucleic acid quantification, demands precise thermal cycling, expensive reagents, and skilled personnel [211], making it less accessible for widespread use. RCA and LCR, despite their innovative amplification strategies, are hindered by prolonged assay times, false positive results, and elevated costs [212]. Moreover, these approaches generally lack the flexibility and simplicity required for integration into portable, point-of-care (POC) diagnostic platforms, which emphasises the need for the development of alternative miRNA detection methods that are rapid, cost-effective, and amenable to real-time and on-site applications. In this direction, biosensors have emerged as excellent platforms for their ability to overcome the aforementioned limitations while ensuring high sensitivity and specificity. The upcoming section focuses on biosensors in diabetes by briefly touching on their historical account, their current status, and market size. Finally, it discusses the scope for new biosensor platforms in diabetes prediction and diagnosis.

## 4. Biosensor in Diabetes

**Figure 4:**
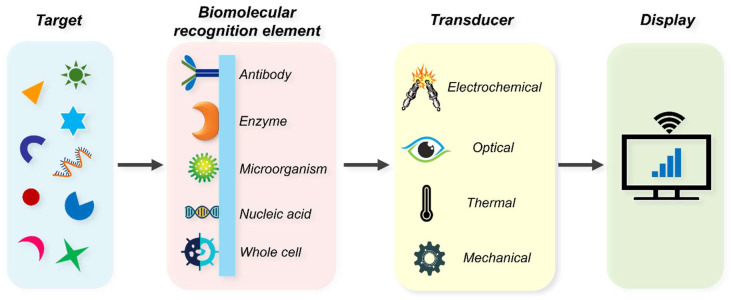
Schematic illustration of a typical biosensor component.

Biosensors have revolutionised the field of diabetes diagnostics, enabling rapid, accurate, and minimally invasive monitoring of blood glucose levels. These devices have evolved from simple enzymatic systems to advanced continuous glucose monitoring technologies, addressing the growing need for effective diabetes management. A biosensor is an analytical device that converts a biological response into a measurable signal [213]. It consists of three primary components: a biomolecular recognition element (MRE), a transducer, and a signal processor (Figure 4). The MRE is often an enzyme, receptor, antibody, aptamer, or nucleic acid that specifically recognises the target analyte [214,215]. The transducer then converts the biological interaction into a measurable physical or chemical signal, such as an electrical current, thermal change, or optical response. Finally, the signal processor interprets this signal into meaningful data, which is displayed to the user. These components work together to enable rapid, accurate, and specific detection, making biosensors indispensable tools in healthcare, particularly in managing chronic conditions like diabetes.

Biosensors can typically be classified into two categories based on the type of MREs and the type of transducer [216]. MREs are the functional components that impart specificity to biosensors by selectively binding target analytes. Common types include enzymes, which catalyse reactions producing measurable signals; antibodies, which enable high-affinity binding for target antigens; and aptamers, synthetic oligonucleotides that offer chemical stability and ease of modification. Nucleic acids are widely used for detecting complementary DNA or RNA sequences. The choice of MRE directly influences sensitivity, selectivity, and operational stability, making their careful selection extremely crucial.

The classification of biosensors based on transducer results in optical (colourimetric, fluorescence, surface plasmon resonance (SPR), chemiluminescence (CL), surface-enhanced Raman scattering (SERS), electrochemiluminescence (ECL), photometric, etc.), electrochemical (potentiometric, voltammetric, photoelectrochemical, conductometric, amperometric, impedimetric, etc.), mechanical (piezoelectric, microcantilevers, etc.), and thermal (calorimetry) biosensors [216,217]. Optical biosensors detect analytes by monitoring changes in light properties, such as absorbance, fluorescence, or refractive index, resulting from specific biorecognition events [218]. Techniques like SPR and localised SPR measure refractive index shifts near a metal surface, allowing real-time, label-free detection of biomolecular interactions. Fluorescence-based sensors exploit emission intensity or lifetime changes upon target binding, while colourimetric assays rely on visible colour changes induced by nanoparticle aggregation or catalytic reactions. Additionally, SERS provides molecular fingerprinting with ultralow detection limits by amplifying Raman signals through plasmonic or semiconductor substrates [218].

Electrochemical (EC) biosensors, on the other hand, measure the electrical signals like current, voltage, or impedance, arising from the analyte-MRE interaction [219]. Among the most widely used techniques, amperometry measures current at a fixed potential, making it suitable for enzyme-based sensors such as glucose oxidase systems. Voltammetry records current as a function of applied potential to provide mechanistic insights into redox processes, while electrochemical impedance spectroscopy (EIS) monitors variations in charge transfer resistance and double-layer capacitance, enabling label-free detection of biomolecular binding events [219].

Mechanical biosensors monitor the physical changes (mass, bending, stretching, etc.) arising from binding between MRE and its cognate analyte [220]. Thermal biosensors work on the principle of measuring heat (absorption or evolution) arising from the bio/chemical reaction [217].

### 4.1. Historical Account of Biosensors in Diabetes

**Figure 5:**
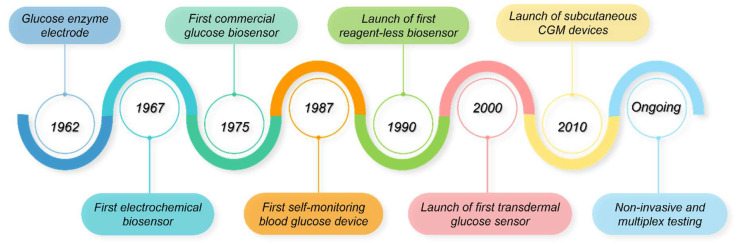
Timeline of major milestones in glucose biosensor development.

The development of biosensors for diabetes management marks one of the most significant advancements in the field of medical diagnostics (Figure 5). In 1962, the invention of the first enzymatic glucose biosensor by Clark and Lyons [221], laid the foundation for glucose monitoring technologies. This pioneering biosensor employed glucose oxidase (GOx) as the MRE to catalyse glucose oxidation and an oxygen electrode as the transducer. The transducer measured the resulting decrease in oxygen concentration levels, providing an indirect measure of glucose concentration. In 1967, building on Clark’s breakthrough work, Updike and Hicks developed the first simplified yet practical glucose biosensor by immobilising and stabilising the GOx in a polyacrylamide gel [222,223]. In 1975, the launch of the first commercial glucose biosensor for direct glucose measurement, the YSI analyser (Model 23A), was initiated by YSI Inc. This device leveraged amperometric detection of hydrogen peroxide (H_2_O_2_), where the electric current generated during the enzymatic oxidation of glucose was directly proportional to glucose concentration [224]. While H_2_O_2_ measurements could be easily achieved on miniaturised devices [225], the high operating potentials required for amperometric H_2_O_2_ measurements led to significant interference from other electroactive species present in biological samples [226]. Moreover, these biosensors were mostly limited to clinical laboratories due to their high costs. To overcome these limitations, redox mediators, such as ferrocene derivatives, thionine, methylene blue, 7,7,8,8-tetracyanoquinodimethane (TCNQ), tetrathiafulvalene (TTF), were introduced to enhance electron transfer, reduce response time, and improve sensor performance [227,228,229]. These biosensors marked the beginning of second-generation biosensors, overseeing the development of commercial screen-printed electrodes in the 1980s [227,230]. The year 1987 saw the launch of ExacTech metre—the first-ever portable EC blood glucose monitor by Medisense Inc. for glucose monitoring [231]. This breakthrough enabled diabetes patients to measure blood glucose levels at home, marking a shift from laboratory-dependent testing to patient-centric management. Of note, even today, the current operation of most commercial glucose sensors is largely similar to that of the ExacTech metre. In the 1990s, third-generation biosensors emerged, which were reagent-less and eliminated the need for redox mediators by using direct electron transfer between the enzyme and electrode [232,233]. In 1999, MiniMed Inc. launched the first commercial in vivo device that provided continuous glucose readings by relying on H_2_O_2_-mediated detection [234]. Subsequent developments focused on miniaturisation, portability, and real-time monitoring of blood glucose. Emergence of continuous glucose monitoring (CGM) systems transformed diabetes management by providing continuous data on glucose levels, alerting users to hyperglycaemia or hypoglycaemia in real-time [235,236,237]. The commercial CGM systems are predominantly EC enzyme-based devices that employ GOx chemistry and rely either on H_2_O_2_ detection or on mediated electron transfer strategies to improve performance.

In this direction, non-invasive glucose biosensor technologies have emerged with the aim of reducing patient discomfort and improving compliance [238]. These devices are commonly based on optical or transdermal approaches. The optical glucose biosensors exploit Raman spectroscopy [239], polarimetry [240], photoacoustics [241], infrared absorption spectroscopy [242], or optical coherence tomography [243] to assess the physical properties of light in the interstitial fluid or the anterior chamber of the eye. The transdermal glucose sensors sit under the skin and track changes in the interstitial glucose levels in real time. The GlucoWatch Biographer by Cygnus Inc. Reference [244] became the first transdermal glucose sensor approved by the US FDA [245]; however, it was never widely accepted in the market due to inherent shortcomings of poor accuracy, false alarm, long warm-up time, and skin irritation. Consequently, it was discontinued in 2008 [245]. Despite this setback, tremendous efforts are being made in developing non-invasive glucose monitoring systems. The Eversense system by Senseonics, for example, uses a fluorescence-based sensor implanted subcutaneously in the upper arm. The sensor is capable of transmitting glucose data for up to 180 days with minimal maintenance [246]. Clinical trials have demonstrated the system to have comparable accuracy to transcutaneous systems, with improved patient satisfaction and reduced perceived distress [247]. Another non-invasive fully implantable CGM system is designed by GlucoTrack^®^ (Rutherford, NJ, USA) that measures glucose directly from the bloodstream. potentially offering greater accuracy and reduced lag time compared to interstitial fluid-based systems. This system potentially offers greater accuracy and reduced lag time compared to interstitial fluid-based systems [248]. These implantable technologies represent a significant advancement in CGM form factors, addressing limitations of wearability, calibration, and sensor longevity. More details on implantable non-invasive CGM systems can be found elsewhere [248,249]. Aside from this, biosensor integration with wireless and smartphone technologies has further improved their usability [250]. Modern CGM devices can transmit glucose data to smartphones, enabling patients and healthcare providers to monitor trends and adjust treatment strategies remotely.

### 4.2. Glucose Biosensor Market

Owing to the increasing prevalence of diabetes across the globe and the demand for more convenient and accurate glucose monitoring methods, the current glucose biosensors market is experiencing significant growth and innovation. According to various reports, the market size is estimated to be valued between USD ~10 billion to ~14 billion, with projections to reach USD 45 billion to USD 50 billion by 2030–2033 [251,252]. This expansion translates to a compound annual growth rate (CAGR) of ~8 to 13% during the forecast period and is indicative of a robust and dynamic market trajectory [251,252].

The rising global burden of diabetes is a primary catalyst for market growth. The increasing prevalence of both T1D and T2D necessitates effective and accessible glucose monitoring solutions. Moreover, growing awareness among patients and healthcare providers regarding the importance of self-monitoring blood glucose (SMBG) for optimal glycaemic control and the prevention of long-term complications is further fuelling the demand for glucose sensors [251]. Technological advancements, particularly in CGM systems, are also pushing market growth for their ability to enable real-time glucose monitoring without the need for repeated fingerstick sampling [252]. This offers patients a significant advantage in overcoming frequent discomfort from finger picking and allows ease of adherence to diabetic management plans. Miniaturisation and improved wearability of the devices are also prominent trends, as smaller devices are more discreet and easier to integrate into daily life [251]. Coupled with this is the seamless integration of smart devices, such as smartphones and smartwatches, which facilitates data sharing, remote monitoring by healthcare professionals, and personalised data analysis for improved diabetes management [251,252].

The classification of the glucose biosensor market based on devices can be broadly segmented into SMBG devices and CGM systems. SMBG represent traditional blood glucose metres requiring finger prick [253], while CGM systems form sensors that are subcutaneously inserted for continuous glucose tracking [254]. The classification based on end-users includes hospitals and clinics, home care settings, and diagnostic centres, each having specific requirements and preferences for glucose monitoring technologies. Geographically, currently, North America holds a dominant market share, attributed to a high prevalence of diabetes and the presence of major industry players within the region [251]. However, the Asia-Pacific region is anticipated to experience the most rapid growth in the coming years. This expansion is anticipated to be driven by the increasing prevalence of diabetes in populous countries like China and India, coupled with rising healthcare expenditure and improved access to advanced medical technologies.

### 4.3. Scope for Potential Biosensors

While glucose biosensors provide a crucial window into real-time glucose fluctuations, they offer a limited perspective on the complex interplay of metabolic processes underlying diabetes and associated conditions. Expanding the scope of biosensing to encompass other emerging biomarkers, as highlighted in Section 4, presents a significant opportunity to gain a more holistic understanding of an individual’s health status and risk of developing complications. Moreover, the integration of glucose monitoring with the assessment of other biomarkers like miRNA and adipokines offers several key advantages. Firstly, monitoring these biomarkers allows for early disease detection and more accurate risk assessment, as changes in these biomarkers precede the changes in glucose levels [50,142]. Secondly, timely interventions exhibit the potential to prevent or delay the onset of diabetes and its associated complications [51]. Moreover, monitoring these biomarkers could facilitate the development of personalised treatment strategies based on the patient’s unique metabolic profile and risk factors. By providing a deeper understanding of the underlying disease mechanisms, these biomarkers can also contribute to the development of novel therapeutic targets [50,51]. Lastly, monitoring these biomarkers exhibits the potential to improve long-term complications prediction, allowing for more proactive management and improved patient outcomes [50]. In view of this, expanding the biosensing technology beyond glucose to include emerging biomarkers like microRNA and adipokines offers a more comprehensive and nuanced approach to diabetes management, moving towards a future of personalised and preventive medicine.

## 5. Nanobiosensors in Diabetes

The amalgamation of sensor technology with nanotechnology has played an important role in developing robust detection systems. These biosensors utilise nanomaterials, including but not limited to metal-based, metal oxide-based, quantum dots (QDs), carbon-based, and metal–organic frameworks (MOFs), to confer high sensitivity, specificity, and detection versatility [255]. Nanomaterials are small materials or chemical substances that possess sizes in the range of 1–100 nm for at least one dimension. They can be classified into different categories based on their morphology, size, composition, and dimensions [256]. The nano-dimensional size of nanomaterials endows them with unique physicochemical properties, such as optical, electrical, thermal, and mechanical characteristics, that are typically absent in bulk materials. The ability of nanomaterials to possess a high surface area-to-volume ratio combined with tunable shape and size and ease of surface functionalisation places nanobiosensors in a different league from conventional biosensors, as these properties enable nanobiosensors with high sensitivity and flexible detection capabilities [255].

### 5.1. Nanobiosensors in miRNA Detection

**Figure 6:**
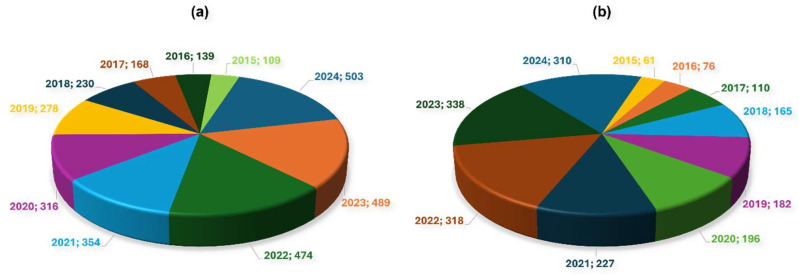
The pie chart shows number of studies published on miRNA detection by employing biosensor (**a**) and nanobiosensor (**b**) platforms in the last ten years (2015–2024). The data was obtained from SCOPUS database by searching keywords microRNA* OR miRNA* AND biosens* for figure (**a**) and microRNA* OR miRNA* AND biosens* AND nano* for figure (**b**).

Over the years, myriad reports investigating the potential of nanobiosensors in miRNA detection have been published (Figure 6). These biosensors commonly employ single-stranded DNA (ssDNA) complementary to the target miRNA as the MRE and utilise nanomaterials with varying shapes, sizes, and compositions to obtain excellent sensitivity and signal amplification. While different detection modalities, including EC, fluorescence, SERS, ECL, photothermal, CL, SPR, and colourimetry, have been developed for miRNA detection (Table 5), most of them transduce the ssDNA-target miRNA hybridisation event. Considering the immense potential of (nano)biosensors in miRNA detection, various reviews have been published in recent years. While some of these reviews present a general overview of different biosensors in miRNA detection [257,258,259,260,261], others specifically focus on either a specific (nano)material type, e.g., QDs [262], magnetic nanoparticles [263], MOFs [264], the specific type of miRNA [265], or the mode of detection, e.g., plasmon-enhanced [266], electrochemiluminescence [267], electrochemical [268,269], and visual [270] biosensors. For the purpose of the review, a brief discussion on different types of nanobiosensors in miRNA detection is presented below.

#### 5.1.1. EC Nanobiosensors in miRNA Detection

**Figure 7:**
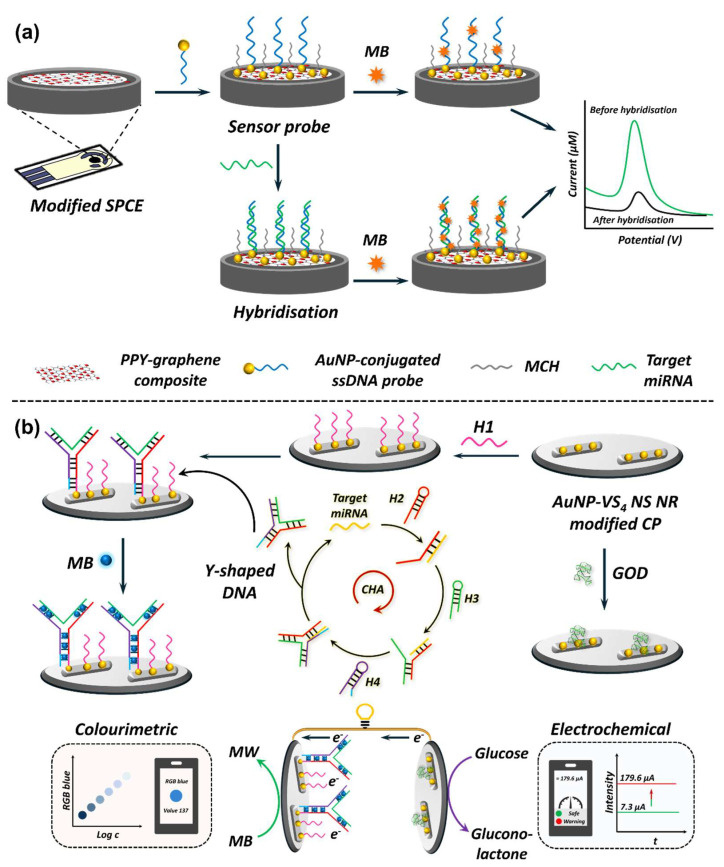
Schematic illustration of EC nanobiosensor fabrication for miRNA sensing using single- (**a**) and dual-mode (EC/colourimetric) (**b**) detection. In single-mode sensor (**a**), a screen-printed carbon electrode (SPCE) modified with polypyrrole (PPY)-graphene nanocomposite, AuNP-conjugated ssDNA probe, and 6-mercapto-1-hexanol (MCH) acts as a sensor probe. When the target miRNA is present, methylene blue (MB) intercalates with the hybridised ssDNA probe-miRNA duplex, generating a strong peak current. In contrast, a significantly smaller peak current is observed in the absence of the target miRNA due to lesser intercalation of MB. The dual-mode sensor (**b**) employs carbon paper (CP) patterned with AuNP-vanadium sulphide nanosheet self-assembled nanorod (AuNP-VS_4_ NS NR) nanocomposite, modified with the H1 probe to function as the EC sensor probe. In parallel, the colourimetric sensor probe is created by modifying the AuNP-VS_4_ NS NR patterned CP with glucose oxidase (GOD). In the presence of target miRNA, a y-shaped DNA generated through catalytic hairpin assembly (CHA) facilitates intercalation of MB, which is then reduced to methylene white (MW) due to the electrons released from the GOD-mediated glucose oxidation. This process results in an amplified open circuit voltage (OCV) signal that can be monitored using a smartphone. The reduction in blue MB into colourless MW enables a colourimetric detection signal, which can also be quantified using a smartphone. Figures are adapted with permission from references [271,292], respectively.

With the success of commercial portable glucose metres, EC biosensors have established their potential as simple, affordable, rapid, and user-friendly platforms. Expectedly, EC biosensors have seen tremendous growth for other targets as well, with miRNAs gaining tremendous attention in the recent past [265,291]. Much like other EC biosensors that measure changes in EC signals arising from bio/chemical events at the electrode surface, the EC miRNA biosensor monitors the hybridisation event between the immobilised ssDNA and cognate target miRNA [268]. Upon hybridisation, change in the EC signal is monitored using techniques like cyclic voltammetry (CV), differential pulse voltammetry (DPV), or EIS [268]. These EC readouts differ in how they probe the electrode interface and thus in their suitability for miRNA assays. CV sweeps the electrode potential and records oxidation/reduction currents, providing mechanistic information about redox-active reporters and surface reaction kinetics, but with modest sensitivity [219]. DPV and square-wave voltammetry (SWV) employ superimposed pulses that suppress capacitive background current, yielding high sensitivity for trace analytes. This makes them methods of choice when a redox reporter or intercalator (e.g., methylene blue, ferrocene) is used for miRNA readout. EIS applies a small alternating current across a range of frequencies to measure interfacial properties such as charge-transfer resistance and capacitance. This technique is particularly valuable for label-free hybridisation assays, as target binding events induce measurable changes in the impedance spectrum, albeit at the cost of longer measurement and more complex data fitting. In practice, CV is primarily a characterisation tool, DPV/SWV are favoured for sensitive, label-based detection, and EIS is preferred for label-free, surface-sensitive miRNA assays. Merits of nanomaterials combined with advancements in nanotechnology have led to the development of single- [292,293] and dual-mode [271,272,273] EC biosensors with sensitivities comparable to gold-standard real-time RT-PCR while maintaining high specificity and rapid turn-around time. In addition, recent efforts have transitioned away from validating only proof-of-concept demonstrations and are focusing on enhancing the sensitivity and target selectivity required to meet clinical standards. The schematics of EC nanobiosensor fabrication using single- and dual-mode miRNA detection are displayed in Figure 7.

Notwithstanding the short duration of miRNAs being considered as potential diagnostic and prognostic biomarkers, EC biosensors have undergone remarkable progress; however, numerous challenges persist. While reports on multiplex miRNA detection have started emerging, most studies commonly focus on single miRNA detection (Table 5), often miR-21, by either synthesising new nanomaterial for electrode modification or signal amplification or combining different amplification techniques to improve biosensor sensitivity [291]. The incorporation of amplification techniques results in higher sensitivity but also contributes to increasing the overall cost, detection time, and complexity of the platform [257]. Specific details on the different target miRNAs tested using electrochemical biosensor and their association with specific health conditions are presented in Table 5. Moreover, nanomaterials used in electrode modification, detection modality (single, dual, or multi-mode), biosensor analytical performance, and validation in simulated/real-clinical samples are also compiled within Table 5.

#### 5.1.2. Optical Nanobiosensors in miRNA Detection

A wide range of optical nanobiosensors, like fluorescence, CL, ECL, SPR, colourimetric, and SERS, have been applied to miRNA detection [218]. These platforms rely on light-based signal transduction but differ in their underlying principles. Fluorescence biosensors detect changes in light emission from fluorophores or nanomaterials. They commonly operate through mechanisms such as fluorescence resonance energy transfer (FRET), where energy is transferred between a donor and an acceptor in close proximity, or through photoinduced electron transfer (PET) and aggregation-induced emission (AIE), depending on the sensor design. CL produces light through chemical reactions, while in ECL, the luminescent species are activated electrochemically at an electrode surface, enabling precise control of signal generation. SPR measures changes in the refractive index near a metal surface when biomolecules bind at the surface, whereas colourimetric biosensors use visible shifts in solution colour, typically from nanoparticle aggregation or dispersion. SERS, in contrast, detects molecular binding events through the enhancement of Raman scattering by plasmonic nanostructures, offering highly specific molecular “fingerprints” with strong sensitivity.

**Figure 8:**
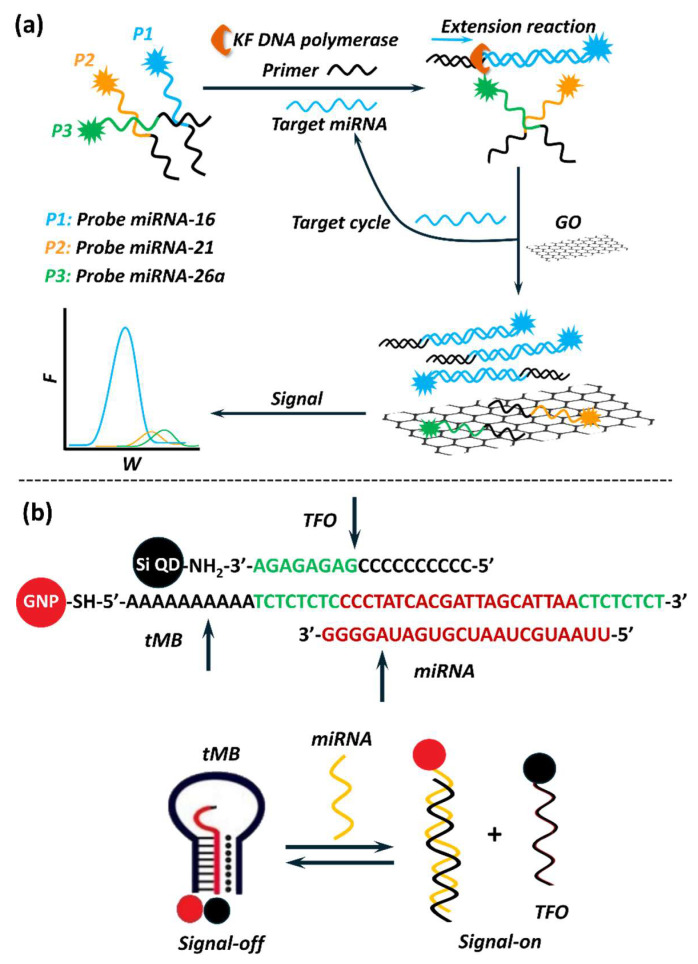
Schematic illustration of fluorescence nanobiosensor fabrication for miRNA sensing using FRET-based approach. Fluorescently labelled DNA probes and graphene oxide (GO) (**a**) serve as donor-quencher pairs and are combined with the isothermal strand displacement amplification (iSDA) technique for the detection of target miRNAs. In the absence of target miRNA, no iSDA occurs, and the signals from all three probes, P1, P2, and P3, remain quenched by GO. When the target miRNA is present, it initiates the amplification process through iSDA, leading to the formation of P1-miRNA duplexes that prevent P1 from adsorbing onto the GO surface (signal-on). Meanwhile, P2 and P3 remain single-stranded and readily adsorb onto the GO surface. This causes their signals to be quenched by FRET (signal-off). In a label-free approach (**b**), silicon quantum dots (Si QDs) and gold nanoparticles (GNPs) were utilised as donor-acceptor pairs. In the absence of the miRNA target, FRET occurs (signal-off) due to the hybridisation between the triplex molecular beacon (tMB) and the triplex-forming oligonucleotide (TFO), bringing the Si QD and GNP into close proximity. However, when the miRNA target is present, the distance between the Si QD and GNP increases due to the hybridisation of tMB with the miRNA target, thereby restoring the Si QD fluorescence signal (signal-on). Figures are adapted with permission from references [276,295], respectively.

Fluorescence nanobiosensors typically rely on fluorescence properties of nanomaterials such as QDs, carbon dots (CDs), graphene oxide (GO), gold nanoparticles (AuNPs), and upconverting nanoparticles (UCNPs), which either act as fluorescent signal emitters or quenchers [265,294]. The mechanism often involves fluorescence resonance energy transfer (FRET), photoinduced electron transfer (PET), or aggregation-induced emission (AIE), depending on the choice of nanomaterial and detection approach [294]. For miRNA detection, these biosensors often employ a hybridisation-based approach, where fluorescently labelled probe DNAs complementary to the target miRNA are immobilised onto or interact with the nanomaterial [257,265]. Upon hybridisation, changes in fluorescence signal occur commonly due to mechanisms like FRET, where energy transfer between the donor and acceptor fluorophores or nanomaterials indicates the presence of the target miRNA. AuNPs [277,279] and GO [295] (Figure 8a) have been widely explored for their quenching potential due to their high surface area and ability to absorb fluorescence energy, creating a “turn-off” state. In the presence of the target miRNA, the fluorescence signal recovers due to hybridisation, creating a “turn-on” state. QDs [276] (Figure 8b) and CDs [296] are gaining popularity in fluorescence biosensor platforms due to their tunable emission spectra and excellent photostability over organic dyes [294]. By employing nanomaterials in fluorescence biosensors, sensitivities ranging from a few nanomolar to femtomolar have been achieved for miRNA detection (Table 5). While some studies employed amplification strategies [279,282,284,285,295] to higher sensitivity, others could achieve similar sensitivity without it [275,276,277,278,280,281,283]. Moreover, excellent recoveries could be obtained in spiked biological samples, indicating their potential for practical application. Specific details on the target miRNA and their implication in particular health conditions, nanomaterials used in biosensor fabrication, mode of detection, biosensor analytical performance, and their validation in simulated/real-clinical samples are discussed in Table 5.

**Figure 9:**
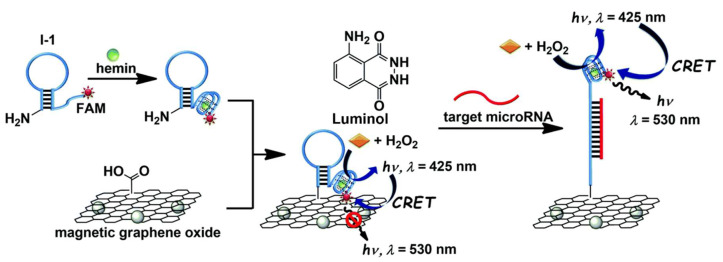
Schematic illustration of CL nanobiosensor fabrication for the detection of miRNA. During the assay, a FAM-labelled hairpin loop (I-1), functionalised with an HRP-mimicking DNAzyme (hemin-intercalated G-quadruplex) serves as the chemiluminescence donor, while magnetic graphene oxide (MGO) functions as the acceptor. In the absence of the target miRNA, the HRP-mimicking DNAzyme/FAM DNA probe remains in a folded conformation, effectively masking the luminescence signal generated by luminol/H_2_O_2_ catalysis via chemiluminescence resonance energy transfer (CRET) (signal-off). However, when the target miRNA is present, the hairpin loop unfolds and hybridises with the target. This interaction increases the distance between the probe DNA and GO, thus preventing CRET (signal-on) and enabling detection of the target. The figure is reproduced with permission from the reference [298].

CL and ECL biosensors have emerged as powerful tools for miRNA detection, offering distinct advantages over traditional fluorescence-based methods [257]. These techniques rely on generating light through chemical or EC reactions, eliminating the need for an external light source and reducing background interference. In CL biosensors, the reaction between a chemical species (e.g., luminol) and an appropriate catalyst produces light emission, which is coupled with miRNA recognition events through amplification strategies [285,297] and/or enzyme-linked reactions [298] (Figure 9). ECL, on the other hand, involves the generation of light through electrochemical stimulation of a luminescent species (e.g., luminol, QDs) at an electrode surface [274,299]. This technique offers higher sensitivity (aM) and control over the reaction compared to CL, as the light emission can be precisely triggered by applying a potential [274]. ECL biosensors can be designed with various configurations, including direct detection of miRNA hybridisation on the electrode surface or indirect detection using labelled probes or nanoparticles [267]. Both CL and ECL-based sensors offer several advantages over fluorescence biosensors. CL and ECL exhibit lower background noise and autofluorescence, i.e., superior signal-to-noise ratio due to the absence of an external light source, leading to improved sensitivity [257]. Furthermore, they circumvent issues associated with photobleaching, thereby resulting in more stable and reliable signals [267]. Photobleaching is a common problem in fluorescence assays that tends to significantly reduce signal intensity over time. However, it should be noted that photobleaching is principally a concern for applications involving repeated or continuous optical excitation and is less problematic for single-use point-of-care assays. Conversely, while CL/ECL overcome light-induced fluorophore degradation, they can face other long-term stability issues like luminophore degradation, electrode fouling, and loss of EC efficiency for long-term or continuous applications. Details on the target miRNAs tested by CL and ECL approach in combination with other approaches, their relation to specific health conditions, nanomaterials used in biosensor fabrication, analytical metrics, and their validation in simulated/real-clinical samples are discussed in Table 5.

**Figure 10:**
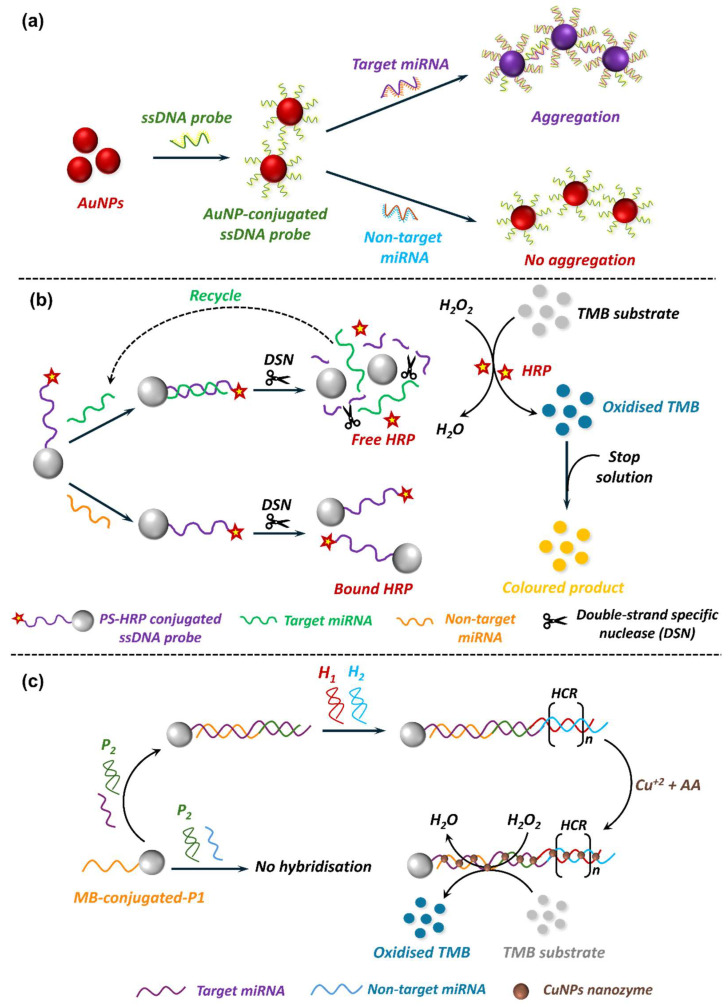
Schematic illustration of colourimetric miRNA detection techniques using AuNP aggregation (**a**), HRP enzyme (**b**), and peroxidase-mimic nanozyme (**c**). In the aggregation approach (**a**), when the target miRNA is absent, the ssDNA probe-functionalized AuNPs remain dispersed, exhibiting a red colour due to the repulsion between negatively charged DNA molecules. However, in the presence of target miRNA, hybridisation occurs between the ssDNA probes and the target miRNA, bringing the AuNPs into close proximity and resulting in aggregation, which produces a blue/purple colour. In the enzyme-based approach (**b**), the presence of target miRNA facilitates the formation of a duplex between polystyrene particles and HRP-conjugated ssDNA probes (PS-HRP-conjugated ssDNA probe). This duplex is recognised by the DSN enzyme, which specifically cleaves the hybridised DNA, leaving the miRNA intact and freeing HRP to catalyse the oxidation of peroxidase substrates (TMB/H_2_O_2_), thereby generating visual signals. In the absence of the miRNA target, the DSN enzyme cannot recognise the PS-HRP-conjugated ssDNA probe, leading to HRP remaining bound and preventing the oxidation of the chromogenic substrate (TMB). In the nanozyme-based approach (**c**), when target miRNA is present, magnetic bead-conjugated DNA probe 1 (MB-conjugated-P1) forms a duplex and subsequently creates a sandwich structure with probe 2 (P2). Hairpins 1 (H1) and 2 (H2) initiate a hybridisation chain reaction (HCR), resulting in the formation of a super sandwich structure on the MBs. The addition of copper salt (Cu^2+^) and ascorbic acid (AA) leads to the generation of peroxidase-mimicking copper nanoparticles (CuNPs), which intercalate into the duplex structure and catalyse the oxidation of the TMB substrate to create a visual signal. Conversely, in the absence of target miRNA, the HCR cannot proceed, preventing Cu^2+^ from interacting with the H1/H2 DNA strands, thus hindering the formation of CuNPs and the subsequent generation of a visual signal. Figures are adapted with permission from references [288,303], and [302], respectively.

Colourimetric biosensors are another type of optical biosensor that have found their application in miRNA sensing due to their simplicity and cost-effectiveness. These biosensors utilise visible colour changes to indicate the presence or absence of target miRNA. The most common strategies for colourimetric miRNA detection involve AuNPs [300], enzyme-based assays [301,302], and nanozyme-mediated [286,288] signal transduction. AuNP-based approaches exploit localised surface plasmon resonance (LSPR) of nanoparticles to enable dramatic shifts in colour in response to aggregation or dispersion [300]. Typically, ssDNA probes complementary to the target miRNA are immobilised onto AuNPs [303]. In the absence of the target miRNA, the AuNPs remain dispersed, retaining their red colour. However, in the presence of target miRNA, hybridisation-induced conformational change or salt-induced aggregation occurs [303,304]. This causes a distinct shift in colour from red to purple or blue, providing a visual readout (Figure 10a). Enzyme-mediated systems employ natural peroxidases like horseradish peroxidase (HRP) to catalyse the oxidation of peroxidase substrates, 3,3’,5,5’-tetramethylbenzidine (TMB) and hydrogen peroxide (H_2_O_2_), to produce visual colour when target miRNA is present (Figure 10b) [301,302]. The biosensor specificity is often achieved by employing ssDNA probes, which hybridise with the target miRNA to induce the desired visual change. Nanozyme-based assays employ nanomaterials exhibiting intrinsic enzyme-mimic catalytic activity to generate a visual signal response [305] (Figure 10c). The incorporation of nanozymes in biosensor platforms has gained momentum for their obvious merits over natural enzymes. Enzymes, being proteins in nature, are prone to denaturation and deactivation under ambient conditions, suffer from expensive and complex production and purification procedures, and are restricted to a narrow temperature and pH range for optimal performance [305,306]. In contrast, nanozymes offer several advantages, including higher stability, tolerance to wider temperature and pH ranges, lower production cost, and catalytic activity similar to, if not better than, natural enzymes [306]. Despite these merits, however, several challenges limit their translation into clinical diagnostics. Unlike natural enzymes, nanozymes often lack substrate specificity, and their catalytic activities can be influenced by non-biological factors, reducing their reliability in complex clinical samples. Moreover, nanozyme activity is highly dependent on synthesis conditions, raising concerns about batch-to-batch reproducibility and scalability for regulatory approval. Thus, while nanozymes show strong promise in proof-of-concept studies, their current performance as catalytic materials has not yet been translated outside controlled laboratory environments, and further work is required to enhance their specificity and clinical reliability.

Colourimetric biosensors, in general, have been reported to achieve nanomolar [307] to picomolar [286] sensitivities for miRNA detection in the absence of amplification techniques. While these assays are promising for their simple and easy-to-use setup, they lack the sensitivity required to meet clinical standards. Despite undergoing overexpression under disease conditions, miRNA level is known to remain within the femtomolar to attomolar range. In view of this, amplification techniques like strand displacement amplification (SDA) [308], HCR [288], HCR with DNAzyme [287], and rolling circle amplification (RCA) with DNAzyme [309] have been utilised to obtain femtomolar to attomolar [288] sensitivity. Recently, efforts have also been directed towards developing sensors that can achieve desirable sensitivity while being enzyme-free as well as amplification-free [310,311]. Specific details on the target miRNA tested using colourimetric nanobiosensors, along with nanomaterials used in sensor fabrication, analytical performance, and their validation in simulated/real-clinical samples, are discussed in Table 5.

### 5.2. Nanobiosensors in Adipokine Detection

T2D is a chronic metabolic disorder that requires early diagnosis and monitoring to prevent the development of severe complications like cardiovascular diseases, neuropathy, retinopathy, and nephropathy. While conventional diagnostic biomarkers highlighted in Section 3.2, like FPG, RBG, 2 h PG, and HbA1c, are widely used, they often fail to provide real-time insights into metabolic dysfunction at an early stage. To this end, adipokines have emerged as promising biomarkers for T2D due to their direct contribution to insulin signalling, inflammation, and energy homeostasis. Over the years, conventional techniques, such as mass spectrometry and ELISA, have been used to measure adipokine levels. Despite offering high sensitivity and specificity, these techniques are limited by their requirements for exhaustive protocols, expensive reagents, trained personnel, sophisticated instruments, and designated laboratory space. Given this, nanobiosensors have emerged as excellent alternatives due to their rapid, highly sensitive, cost-effective, and user-friendly approach to adipokine detection. The high specificity is ensured by using antibodies or aptamers as MREs.

In recent years, nanomaterials, including metal nanostructures (e.g., AuNPs [312,313,314], gold nanotriangles (AuNTs) [315], gold nanorods (AuNRs) [316], silver nanowires (AgNWs) [317]), metal oxide nanostructures (e.g., zinc oxide nanorods (ZnO NRs) [318], zinc oxide nanoparticles (ZnO NPs) [319], iron oxide nanoparticles (Fe_3_O_4_/Fe_2_O_3_ NPs) [320], titanium dioxide nanoparticles (TiO_2_NPs) [321]), bimetal nanoparticles (e.g., iron-nickel nanoparticles (FeNi NPs) [322], Fe_3_O_4_ core and Au shell (Fe_3_O_4_@Au) [323], Au core and Ag-Au alloy shell (Au@Ag-Au) [324]), carbon-based nanomaterials (e.g., reduced GO (rGO) [325], graphene [326]), and others (e.g., MOFs [327], UCNPs [328], etc.) have been employed in developing biosensors for adipokine detection. So far, detection modalities, such as EC, colourimetric, fluorescence, SERS, SPR, CL, photothermal, etc., have been explored for adipokine detection; however, EC remains the most exploited mode. The following section focuses on recent developments in nanobiosensors for adipokine detection using the aforementioned modes.

#### 5.2.1. EC Nanobiosensors in Adipokine Detection

**Figure 11:**
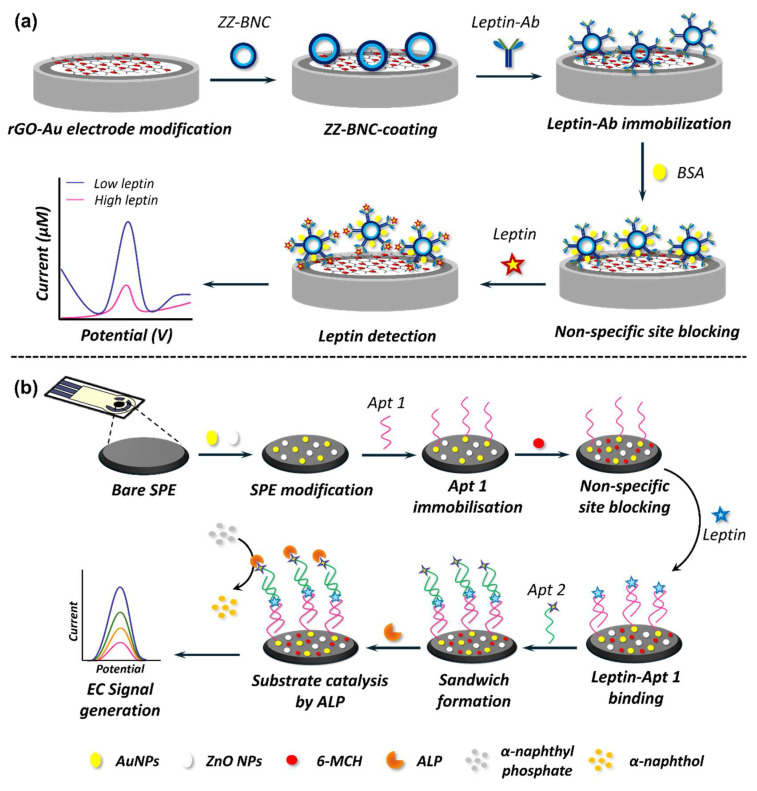
Schematic illustration of the fabrication of EC antibody- (**a**) and aptamer-based (**b**) nanobiosensor for leptin detection. In antibody-based biosensor (**a**), a reduced graphene oxide (rGO)-Au nanocomposite patterned electrode is modified with bionanocapsule-ZZ (ZZ-BNC) and bovine serum albumin (BSA). The rGO-Au patterning ensures signal amplification, while ZZ-BNC facilitates the vertical orientation of antibodies, and BSA prevents non-specific interactions at the electrode surface. In the presence of leptin, the binding between leptin and the antibody hinders efficient electron transfer due to increase in charge transfer resistance, thereby causing a decrease in the EC signal. Conversely, in the absence of leptin, electron transfer occurs efficiently, resulting in a high EC signal. In the aptamer-based biosensor (**b**), a pair of aptamers is employed to create a sandwich-type biosensor. Zinc oxide nanoparticles (ZnO NPs), AuNPs, and capture aptamers (Apt 1) are utilised to modify the screen-printed electrode (SPE), while alkaline phosphatase (ALP)-labelled aptamers (Apt 2) serve as detection aptamers. When leptin is present, a sandwich complex forms that traps leptin between the capture and detection aptamers. This results in ALP converting the inactive α-naphthyl phosphate substrate into the electroactive product α-naphthol, producing a strong current signal. In the absence of leptin, however, this sandwich complex does not form on the electrode surface, inhibiting the generation of the electroactive product and resulting in weak current signals. Figures are adapted with permission from references [319,325], respectively.

Owing to their inherent sensitivity, EC nanobiosensors have emerged as powerful analytical platforms for adipokine detection. The operational principles of EC biosensors are diverse and are often tailored to the specific requirements of the target adipokine and the intended application. The amperometric detection, for instance, is suitable when the analyte or a reporter molecule undergoes a redox process, facilitating rapid electron exchange and yielding real-time data [329]. Voltammetric techniques are ideal for detecting subtle changes in the EC profile induced by adipokine binding events, particularly when the sensor surface is modified with nanomaterials to promote electron transfer [319,325,326,330]. Meanwhile, in the EIS method, the binding of an adipokine typically leads to alterations in charge transfer resistance and capacitance, enabling detection at ultra-low concentrations [321,322,331]. To confer adipokine specificity in these EC biosensors, antibodies have long served as the primary MRE due to their inherent high affinity and selectivity for target adipokine. These sensors utilise immobilised antibodies on the electrode surface, where measurable EC changes are observed in response to the specific adipokine binding. For instance, antibody-functionalised electrodes have been successfully employed for the sensitive detection of leptin [322,325,326,330] and TNF-α and IL-6 [329] simultaneously. Combining nanomaterials with antibodies ensured fg/mL LOD towards leptin (Figure 11a), while pg/mL LOD was obtained for TNF-α and IL-6. Despite the obvious merits of antibodies, their imitations of poor stability and high production and purification costs have led to the development of alternative MREs. One such example is aptamers, single-stranded DNA or RNA oligonucleotides that fold into unique three-dimensional structures to bind with their targets. Being commonly DNA in nature, these aptamers exhibit significant advantages over antibodies, such as high stability, inexpensive synthesis, and ease of tunability, while exhibiting high target specificity. For instance, aptamer-functionalised electrodes have been successfully employed for leptin [319,321] (Figure 11b) and IL-6 [331] detection, yielding fg/mL and pg/mL detection limits for leptin and IL-6, respectively. Table 6 compiles examples of various adipokines detected using EC nanobiosensors and highlights their specific details, including health condition, nanomaterial used in electrode modification, sensor analytical performance, and MRE. It also provides information on the practical applicability of the biosensor platform.

While EC nanobiosensors exhibit promising attributes for adipokine detection, several critical limitations hinder their widespread clinical and research adoption. One significant challenge is the reproducibility of sensor fabrication arising from batch-to-batch variability in nanomaterial synthesis. This can lead to inconsistent sensor performance and compromise its quantitative accuracy. The complexity of biological matrices such as serum or plasma presents issues of non-specific adsorption and interference from other electroactive species, which may result in increased background noise and reduced specificity. The long-term stability and durability of these sensors also remain problematic, as electrode fouling and the degradation of MREs can limit sensor lifespan and operational reliability.

#### 5.2.2. Optical Nanobiosensors in Adipokine Detection

**Figure 12:**
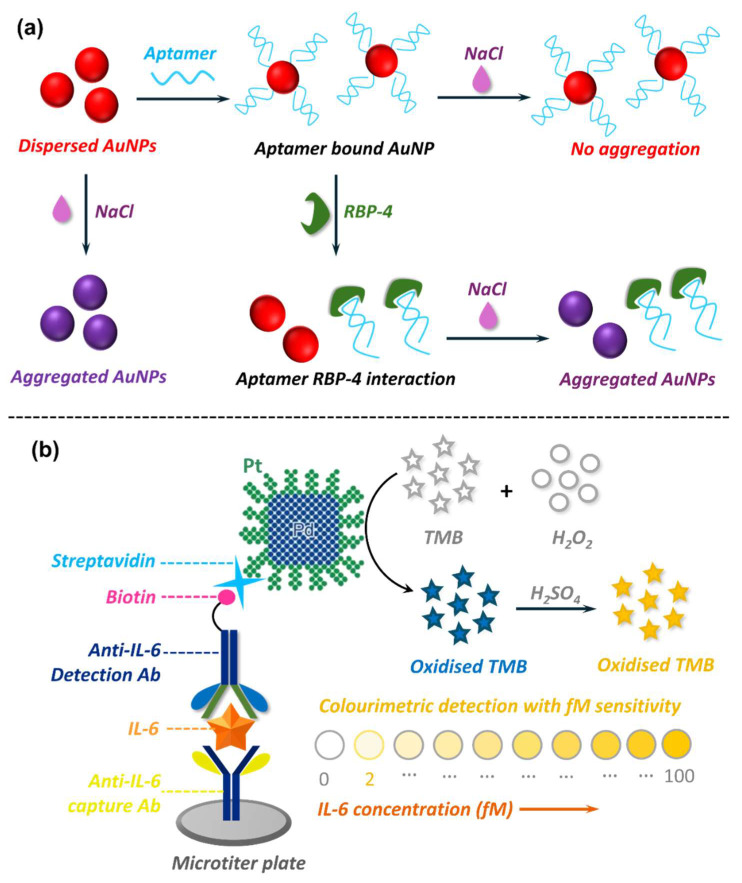
Schematic illustration of colourimetric nanobiosensor fabrication for RBP-4 (**a**) and IL-6 (**b**) detection. For RBP-4 detection (**a**), aptamer-functionalised AuNPs are utilised as sensor probes. In the presence of RBP-4, the aptamers desorb from the AuNP surface to interact with the target, leaving the AuNP surface unprotected against salt-induced aggregation (purple colour). Conversely, in the absence of RBP-4, the aptamers remain attached to the AuNPs, protecting them from aggregation and, hence, exhibiting a red colour. For IL-6 detection (**b**), a sandwich-type ELISA was developed by incorporating anti-IL-6 as the capture antibody and peroxidase-mimicking Pd@Pt core–shell nanodendrites-modified anti-IL-6 as the detection antibody. In the presence of IL-6, a sandwich complex is formed, trapping IL-6 between the capture and detection antibodies. This leads to peroxidase nanozyme-mediated TMB/H_2_O_2_ catalysis, resulting in a visual signal that correlates to the IL-6 concentration. In contrast, when the target is absent, the sandwich complex does not form, preventing the detection antibody from binding to the microtiter plate. As a result, TMB/H_2_O_2_ catalysis is inhibited, and no visual signal is produced. Figures are adapted with permission from references [312,333], respectively.

In recent years, optical detection modalities, including colourimetric, fluorescence, SERS, CL, and SPR, have been investigated for adipokine detection. Colourimetric nanobiosensors for adipokine detection operate by inducing a visible colour change upon their binding, often through AuNP aggregation [312,314], enzyme-linked [335], or nanozyme-based [327,333] reactions. In the aggregation-based approach, AuNPs are functionalised by adipokine-specific aptamers using covalent [314] or non-covalent [312] interactions. In the presence of target adipokine (RBP-4), the non-covalently bound aptamers desorb from the AuNP surface, leaving the nanoparticle exposed to salt-induced aggregation, hence the visible colour change [312] (Figure 12a). In the case of covalent functionalisation, aptamer pairs are used to form a sandwich format and the presence of adipokine (IL-6) is confirmed by the change in colour due to AuNP aggregation [314]. The enzyme-based method also employed a sandwich format; however, it used antibody-pair, i.e., capture-detection antibodies. The capture antibodies were functionalised onto magnetic beads, while detection antibodies were labelled with HRP. In the presence of target IL-6, a sandwich formation occurs, enabling HRP to oxidise the TMB substrate using H_2_O_2_, which is further oxidised to produce visible colour using H_2_SO_4_ [335]. The nanozyme-based system employs a similar approach for IL-6 detection with minor changes [333]. In this case, the HRP enzyme was replaced with peroxidase-mimic Pd@Pt nanodendrites (Pd@Pt NDs) (Figure 12b), and the rest of the assay was kept the same. Table 6 compiles examples of various adipokines detected using colourimetric nanobiosensors and highlights their specific details, including health conditions, nanomaterials used as labels or signal amplifiers, sensor analytical performance, and MRE. It also provides information on the practical applicability of the biosensor platform.

**Figure 13:**
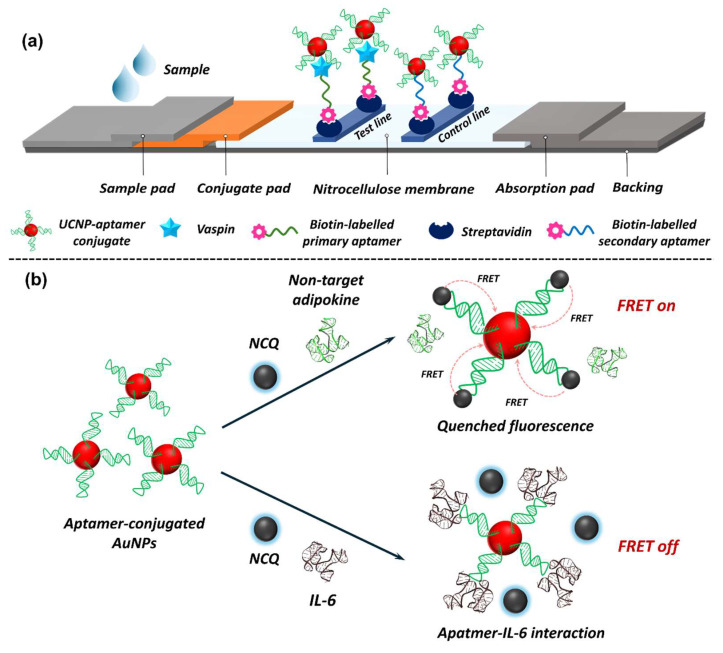
Schematic illustration of fluorescence-based nanobiosensor fabrication for vaspin (**a**) and IL-6 (**b**) detection. Vaspin detection is mediated on a sandwich-format lateral flow device (LFD) by utilising UCNPs as fluorescence labels and aptamers as MREs. The UCNP-aptamer conjugates are pre-immobilised on the conjugate pad. In the presence of vaspin, these conjugates form a UCNP-aptamer-vaspin complex, which is then captured by biotin-labelled primary aptamers at the test line, creating a sandwich structure. Unbound or excess UCNP-aptamer conjugates are captured by biotin-labelled secondary aptamers at the control line. Fluorescence signals at both the test and control lines indicate the presence of vaspin, while a lack of signal at the test line confirms its absence. IL-6 detection (**b**) was facilitated using FRET on/off mechanism, wherein nitrogen-doped carbon quantum dots (NCQ) act as donors and aptamer-conjugated AuNPs as acceptors. In the absence of IL-6 or the presence of non-target adipokine, the NCQ is able to interact with aptamers via π-π binding and their fluorescence is quenched by AuNPs (FRET on). However, in the presence of IL-6, the aptamers bind to the target IL-6, preventing NCQ adsorption and allowing their fluorescence to persist (FRET off), with the intensity corresponding to the concentration of IL-6. Figures are adapted with permission from references [328,332], respectively.

While colourimetric nanobiosensors are simple, user-friendly, and instrument-free, their wider application is often restricted due to their limited sensitivity. Given this, fluorescence nanobiosensors are being developed for adipokine detection. These biosensors rely on the emission of light from UCNPs [328], carbon QDs [332] or fluorescent tags [318] to enable vaspin (Figure 13a), IL-6, and TNF-α detection, respectively. Despite using different MREs, aptamers for vaspin and antibodies for TNF-α, both sensors relied on the sandwich format to mediate adipokine target detection, enabling sensitivities in the range of pg/mL to fg/mL. FRET-based detection is another approach to mediate adipokine detection, wherein the sensor operates on the FRET on/off mechanism by utilising aptamer as MRE and carbon QDs and AuNPs as donor-quencher pair [332] (Figure 13b). In the absence of target IL-6, AuNPs functionalised with aptamers quench the QD fluorescence by FRET, rendering them in ‘FRET on’ the state. However, no such quenching is evident in the presence of the target due to the increased distance between the donor and quencher pair, resulting in a ‘FRET off” state. More details of these examples, including the implication of adipokine in specific health conditions, nanomaterials employed in sensor development, analytical performance metrics, and type of MRE, are summarised in Table 6. Moreover, information on the practical applicability of the biosensor platform is also highlighted depending on whether sensor performance was validated in real biological samples. Albeit fluorescence biosensors hold tremendous potential, only a handful of reports have emerged in adipokine detection, which indicates the need for extensive efforts in this direction. Moreover, most of these adipokines are associated with health conditions beyond diabetes, which, while being useful, cannot be directly expected to accurately analyse adipokines in diabetic samples as their levels tend to vary depending on disease conditions.

Other less commonly explored optical detection modes in adipokine sensing include SPR and SERS. SPR-based biosensors rely on the principle of plasmonic resonance, where changes in refractive index at the sensor surface alter the resonance angle of incident light due to biomolecular interactions. Recently, AuNRs [316] and magnetic nanoparticles (MNPs) [320] have been exploited for their application in SPR biosensors for adipokine detection. The AuNR-based sensor utilised adipokine-specific antibodies to enable simultaneous detection of TNF-α, IL-6, and TGF-β with pg/mL sensitivity [316]. In contrast, the MNP-based sensor detected RBP-4 along with other cytokines, neutrophil gelatinase-associated lipocalin (NGAL) and interleukin-18 (IL-18) with ng/mL sensitivity, using antibody-pair in a sandwich format [320]. Apart from the simultaneous detection of three clinically relevant biomarkers, both sensors displayed potential for practical applicability by detecting targets in real biological matrices. More details of these examples, including the implication of adipokine in specific health conditions and biosensor analytical performance, are summarised in Table 6.

SERS nanobiosensors offer ultrasensitive adipokine detection by exploiting the plasmonic enhancement of Raman signals by metal nanostructures such as AuNTs [315] and Au@Ag-Au core–shell [324] nanoparticles. The underlying detection principle of these biosensors was based on the amplification of vibrational spectra due to the adsorption of adipokine onto these plasmonic substrates, thereby enabling label-free detection of adiponectin [315] and IL-6 [324] with fg/mL sensitivity. In both biosensors, high specificity was conferred by the functionalisation of the nanostructure surface with antibodies, while Raman reporter molecules facilitated the acquisition of distinct spectral fingerprints upon adipokine binding. More details of these examples, including the implication of adipokine in specific health conditions and biosensor analytical performance, are summarised in Table 6.

Despite the tremendous potential of SPR and SERS-based nanobiosensors in adipokine detection, there remains a significant lack of efforts to detect these biomarkers. This lacuna can potentially be attributed to the challenges associated with the respective platforms. For instance, both SPR- and SERS-based biosensors may suffer from poor sensitivity in analysing trace analytes in complex biological matrices and may undergo non-specific binding, compromising sensor performance [336]. Another potential limitation of these biosensors is that MREs may lose their native configuration during immobilisation on the sensor chip surface, or their orientation may sterically hinder the analyte binding [336]. Similarly, SERS nanobiosensors can suffer from reproducibility issues due to batch-to-batch variability in nanomaterial fabrication [337]. In complex biological matrices, nanomaterials may undergo undesirable morphological changes due to aggregation or non-specific biomolecule adsorption, causing loss of signature SERS signals [337]. Additionally, both platforms may experience difficulties in maintaining sensor stability and specificity in heterogeneous samples and face challenges related to surface fouling and surface functionalisation required to ensure selective adipokine binding. These limitations highlight the need for continued optimisation in nanofabrication techniques and surface chemistry to fully realise the potential of SPR and SERS in clinical adipokine diagnostics.

## 6. Challenges and Future Perspectives

Although nanobiosensor technologies have significantly advanced the detection of circulating miRNAs and adipokines, several critical challenges must be addressed to translate these innovations into clinical practice for diabetes diagnosis and monitoring.

Currently, the majority of nanobiosensor platforms target miRNAs associated with oncological diseases, with limited efforts directed toward diabetes-related miRNAs. Furthermore, most studies focus on the detection of individual miRNAs, such as miR-21 or miR-155, while only a limited number investigate the multiplex detection of miRNAs within a unified sensor platform. It is crucial to highlight that individual miRNAs, such as miR-21, may be implicated in various disease conditions, thereby rendering the detection of a single miRNA insufficient for definitive diagnosis of any particular health condition. The development of multiplexed biosensing platforms capable of detecting panels of diabetes-specific miRNAs and adipokines simultaneously is essential for improving diagnostic accuracy and capturing the complex biomolecular signatures associated with disease progression.

Under pathological conditions, miRNA levels undergo either over- or under-expression, yet their circulating concentrations generally fall within the femtomolar to attomolar range. Consequently, amplification strategies such as RCA, SDA, and HCR are employed to enhance sensitivity. While these techniques improve detection capabilities, they inadvertently increase assay complexity, response time, and overall costs, which counteracts the primary objective of developing simple, rapid, and affordable biosensor platforms.

In the domain of adipokine detection, current efforts predominantly rely on antibody-based biosensors integrated into EC platforms. While antibodies offer excellent target specificity, their inherent instability under ambient conditions and high production costs pose substantial limitations. Antibody-derived fragments, including single-chain variable fragments (ScFv) and antigen-binding fragments (Fab), represent promising alternatives. These fragments retain high target specificity while offering advantages such as smaller size, which can improve target accessibility and reduce steric hindrance, and potentially enhanced stability under various conditions. Despite these benefits, challenges remain in their consistent large-scale production, reliable functionalization, and long-term stability, which must be addressed to enable their practical implementation in next-generation biosensing platforms. Aptamer-based biosensors provide a promising alternative with advantages in stability, cost, and ease of synthesis; however, only a limited number of adipokine-specific aptamers have been developed, and their integration into nanobiosensing platforms for diabetes biomarkers remains in its infancy.

Beyond analytical performance, manufacturability represents a major bottleneck in the clinical translation of these nanobiosensors. The ability to design sensors with consistent quality across batches is essential for achieving reliable calibration, securing regulatory approval, and ensuring reproducible clinical performance. Many promising sensors remain confined to proof-of-concept demonstrations because they cannot be manufactured reliably at scale, which ultimately limits their clinical utility. Incorporating design-for-manufacturing principles early in development, such as standardised fabrication processes, robust material selection, and scalable assembly, can significantly enhance the likelihood of successful translation.

Future research should focus on expanding the repertoire of diabetes-specific miRNAs and adipokines targeted by nanobiosensors, alongside the development of robust multiplex detection platforms. Innovative signal amplification strategies that maintain simplicity and cost-effectiveness must be prioritised to enhance sensitivity without compromising practicality. Furthermore, the exploration of enzyme-free biosensors, such as those utilising nanozymes, offers a promising avenue, though this field is still in its early stages. Significant work is also needed to improve the clinical validation of these technologies through large-scale studies using real patient samples, ensuring reliability and reproducibility in diverse clinical settings.

Ultimately, overcoming these challenges will require a multidisciplinary approach that bridges advances in materials science, molecular biology, and clinical diagnostics. The successful development of next-generation nanobiosensors capable of detecting miRNAs and adipokines with high sensitivity, specificity, ease-of-use, and manufacturability has the potential to transform the early diagnosis and personalised management of diabetes and its complications.

## Figures and Tables

**Table 1:** Diagnostic criteria for diabetes and prediabetes.

Condition	ADA Criteria [37]	WHO Criteria [44]
**Normal**	FPG ≤ 100 mg/dL (≤5.5 mmol/L)	
	2 h PG during OGTT ≤ 140 mg/dL (≤7.7 mmol/L)	
	HbA1c ≤ 5.7%	
**Diabetes**	FPG ≥ 126 mg/dL (≥7.0 mmol/L)	
	RBG ≥ 200 mg/dL (≥11.1 mmol/L)	
	2 h PG during OGTT ≥ 200 mg/dL (≥11.1 mmol/L)	
	HbA1c ≥ 6.5%	
**Pre-diabetes**	FPG 101–124 mg/dL (5.6–6.9 mmol/L)	FPG 110–124 mg/dL (6.1–6.9 mmol/L)
	HbA1c 5.7–6.4%	HbA1c 6.0–6.4%
	2 h PG during OGTT 140–198 mg/dL (7.8–11.0 mmol/L)	

**Table 3:** Emerging biomarkers in T2D.

Biomarker Type	Examples	Role in T2D	Reference
Glucose metabolism biomarkers	HbA1c, fasting glucose, glycated albumin, fructosamine, 1,5-AHG	Markers of impaired glucose metabolism and progression to T2D.	[140,145,146,147,148]
Insulin-Related biomarkers	Fasting insulin, C-peptide, HOMA-IR, proinsulin	Indicators of insulin resistance and beta-cell dysfunction.	[149,150,151,152,153]
Metabolites	BCAAs, acylcarnitines, tyrosine, phenylalanine, glycine	Indicators of altered metabolic pathways related to glucose and lipid metabolism.	[154,155,156,157,158]
Lipid biomarkers	Triglycerides, HDL, LDL, free fatty acids, lipoprotein(a)	Markers of dyslipidaemia and metabolic syndrome.	[159,160,161,162,163]
Genetic biomarkers	*TCF7L2, FTO, PPARG, KCNJ11, SLC30A8, CDKN2A/B, TOX, HHEX, IGF2BP2*	Variants in genes associated with β-cell function, insulin sensitivity, and glucose metabolism.	[159,160,161,162,163,164,165,166]
Inflammatory biomarkers	CRP, IL-6, TNF-α, IL-1β, MCP-1	Indicators of chronic inflammation linked to insulin resistance.	[167,168,169,170,171,172]
Gut Microbiota	Reduced *Akkermansia muciniphila*, increased *Firmicutes*/*Bacteroidetes* ratio, reduced butyrate levels	Patterns of gut microbiota composition linked to glucose metabolism and inflammation.	[173,174,175,176,177]
Proteomic biomarkers	Fetuin-A, ceruloplasmin, FGF21, SHBG	Indicators of protein-related pathways influencing glucose metabolism and insulin resistance.	[178,179,180,181]

**Table 4:** The classification of adipokines and their association with T2D.

Classification	Adipokine	Function	Association with T2D	Reference(s)
Anti-inflammatory	Adiponectin	Enhances insulin sensitivity, reduces inflammation, and promotes fatty acid oxidation.	Reduced levels are associated with insulin resistance and T2D.	[186,187]
	Vaspin	Inhibits pro-inflammatory cytokines, enhances insulin sensitivity, and regulates metabolic homeostasis.	Elevated levels correlate with obesity and impaired insulin sensitivity	[188,189]
	Omentin-1	Improves insulin sensitivity, modulates glucose metabolism, and suppresses inflammation.	Reduced levels correlate with insulin resistance and inflammation.	[190]
	Gremlin-1	Improves insulin sensitivity, promotes metabolic homeostasis; emerging therapeutic target.	Increased levels correlate with obesity and insulin resistance.	[191]
	FGF-21	Regulates glucose and lipid metabolism, reduces inflammation, and protects against metabolic stress.	Increased levels are linked to metabolic dysfunctions in T2D.	[192,193]
	Leptin	Regulates appetite and energy balance; elevated levels linked to chronic inflammation in obesity.	Increased levels are associated with obesity and leptin resistance.	[194]
Pro-inflammatory	Resistin	Promotes insulin resistance, increases pro-inflammatory cytokine production, and links obesity to T2D.	Elevated levels are associated with an increased risk of T2D.	[195,196]
	Chemerin	Increases inflammation, regulates adipocyte differentiation, and promotes insulin resistance.	Elevated levels are linked with metabolic syndrome and T2D.	[197,198]
	Visfatin	Enhances inflammatory responses and may modulate insulin secretion.	Elevated levels are observed in metabolic syndrome and T2D.	[199]
	RBP-4	Induces insulin resistance, links adipose dysfunction to glucose intolerance, and promotes inflammation.	Elevated levels are strongly associated with insulin resistance.	[200]
	TNF-α	Promotes systemic inflammation and contributes to insulin resistance in obesity and T2D.	High levels are linked with systemic inflammation and insulin resistance	[201]
	IL-6	Induces chronic low-grade inflammation, impairs insulin signalling and metabolic regulation.	Elevated levels (>3 pg/mL) correlate with systemic inflammation and T2D.	[202]

**Table 5:** Nanobiosensor-based detection of miRNAs under different disease conditions ^1^.

miRNA	Health Condition	Mode of Detection and Technique	Nanomaterial(s)	Analytical Performance	Remarks	Reference
miR-21	Lung cancer	EC using OCV/ colourimetric dual-mode	VS_4_ NS-NR-AuNP hybrid nanocomposite	LR = 0.001–10^3^ pg/mL LOD = 0.16 fg/mL Recovery = 84.1–100.6%	Required amplification = no Real sample validation = yes	[271]
miR-21	Cancer	EC using OCV/ colourimetric dual-mode	Graphdiyne/AuNPs composites	LR = 0.5 fM–100 pM LOD = 0.15 fM (EC) 33 fM (Colourimetric) Recovery = 97.6–105.7%	Required amplification = yes Real sample validation = yes	[272]
miR-141	Breast cancer	EC using OCV/ colourimetric dual-mode	Graphdiyne/AuNPs composites	LR = 0.0001–100 pM LOD = 21.9 aM Recovery = 99.0–106.3%	Required amplification = no Real sample validation = yes	[273]
miR-141	Cancer	ECL/SPC dual-mode	S-BN QDs and WO_3-x_ nanodots	LR = 10^−17^–10^−10^ M LOD = 10^−17^ M Recovery = 99.0–103%	Required amplification = no Real sample validation = yes	[274]
miR-21	Ovarian cancer	Fluorescence	AgNCs	LR = 9 pM–1.55 nM LOD = 2 pM Recovery = 93–108%	Required amplification = no Real sample validation = yes	[275]
miR-155	Cancer	Fluorescence	Si QDs and AuNPs	LR = 10–800 pM LOD = 10 pM Recovery = 99–100.2%	Required amplification = no Real sample validation = yes	[276]
miR-155	Cancer	Fluorescence	MSN@R6G @AuNP	LR = 100 fM–100 nM LOD = 2.18 fM Recovery = 99–100.2%	Required amplification = no Real sample validation = yes	[277]
miR-155	Cancer	Fluorescence	La(III)-MOF and AgNPs	LR = 2.7 fM–0.01 pM LOD = 5.2 fM Recovery = NR	Required amplification = no Clinical sample validation = yes	[278]
miR-125a-5p	Acute ischemic stroke	Fluorescence	AuNPs	LR = 0.01–10 µM LOD = NR Recovery = 100.6–107.4%	Required amplification = yes Real sample validation = yes	[279]
miR-222	Cancer	Fluorescence	UCNPs	LR = 0.5–2.5 nM LOD = 0.077 nM Recovery = 97.6–102.1%	Required amplification = no Real sample validation = yes	[280]
miR-362	Inflammatory bowel disease	Fluorescence	AgNCs	LR = 20–200 nM LOD = 6.5 nM Recovery = 74.7–92.8%	Required amplification = no Real sample validation = yes	[281]
miR-499	Acute myocardial infarction	Fluorescence	MoS_2_ NS	LR = 0.1–13.33 nM LOD = 381.8 pM Recovery = 89.5–97.6%	Required amplification = yes Real sample validation = yes	[282]
miR-21 and miR-155	Cancer	Fluorescence	QDs	LR = 0.1 pM–0.01 nM (both miRNA) LOD = 0.1 pM (both miRNA) Recovery = NR	Required amplification = no Clinical sample validation = yes No labelling required	[283]
miR-21 and miR-155	Breast and lung cancer	Fluorescence/ photothermal dual-mode	CeO_2_@AuNP	LR = 10 pM–100 nM (miR-21) and 10 pM–200 nM (miR-155) LOD = 7.2 pM (miR-21) and 9.1 pM (miR-155) Recovery = NR	Required amplification = yes Clinical sample validation = yes Nanozyme-based detection	[284]
miR-21 and miR-155	Cancer	Fluorescence (FL)/CL dual-mode	AuNPs	LR = 500 fM–10 nM (FL) 10 fM–10 nM (CL) for both miRNAs LOD = 107.2 fM (FL), 8.3 fM (CL) for miR-21 and 97.7 fM (FL), 11.2 fM (CL) for miR-155 Recovery = 93.4–121.8% (FL) 87.4–110.6% (CL) for miR-155	Required amplification = yes Real sample validation = yes	[285]
miR-21	Cancer	Colourimetric	Ag/PtNCs	LR = 10–1000 pM LOD = 4.1 pM Recovery = 93.8–106%	Required amplification = no Real sample validation = yes Nanozyme-based detection on µPAD	[286]
miR-21	Cancer	Colourimetric	Fe_3_O_4_ NPs	LR = 5 fM–10 pM LOD = 5 fM Recovery = 97.5 –104%	Required amplification = yes Real sample validation = yes	[287]
miR-155	Cancer	Colourimetric	CuNPs	LR = 80–500 aM LOD = 22 aM Recovery = 98.8–103.4%	Required amplification = yes Real sample validation = yes Nanozyme-based detection	[288]
miR-155	Cancer	Colourimetric	AuNPs	LR = 1–100 nM LOD = 0.7 nM Recovery = 96.8–103%	Required amplification = no Real sample validation = yes	[289]
miR-148a	Gastric cancer	Colourimetric	AuNPs	LR = NR LOD = 1.9 nM Recovery = NR	Required amplification = no Real sample validation = no	[290]

^1^ This table is not exhaustive and only compiles studies published in the last five years, 2019–2024.

**Table 6:** Nanobiosensor-based adipokine detection under different disease conditions ^1^.

Adipokine	Health Condition(s)	Mode of Detection and Technique	Nanomaterial	Analytical Performance	Remarks	Reference
Leptin	Obesity	EC using EIS	FeNi NPs	LR = 0.5 pg/mL–80 ng/mL LOD = 157.4 fg/mL Recovery = NR	Antibody-based detection. Real sample validation matrix was mouse serum.	[322]
Leptin	Neuro-degenerative disease and brain trauma	EC using DPV	ZnO NPs and AuNPs	LR = 0.01–100 pg/mL LOD = 0.035 pg/mL Recovery = 96.3–103.4% (serum) 97.8–108.8% (plasma)	Aptamer pair-based detection. Real sample validation matrix was serum and plasma.	[319]
Leptin	Traumatic brain injury	EC using EIS	TiO_2_ NPs and AuNPs	LR = 1–100 pg/mL and 100–1000 pg/mL LOD = 0.31 pg/mL Recovery = 96.4–107.8%	Aptamer-based detection. Real sample validation matrix was serum and plasma.	[321]
Leptin	Obesity-related conditions	EC using DPV	rGO and AuNPs	LR = 0.001–1000 pg/mL LOD = 0.87 fg/mL Recovery = NR	Antibody-based detection. Real sample validation matrix was mice and human serum	[325]
Leptin	Non-alcoholic fatty liver	EC using SWV	Porous graphene functionalised black phosphorous and AuNPs	LR = 0.15 pg/mL–2.5 ng/mL LOD = 0.036 fg/mL Recovery = 98–100.8%	Antibody-based detection. Real sample validation matrix was serum.	[326]
Leptin	Obesity	EC using DPV	Ce_3_NbO_7_/CeO_2_ hollow nanospheres	LR = 0.5 pg/mL– 12 ng/mL LOD = 0.14 fg/mL Recovery = 86.05–94.7%	Antibody-based detection. Real sample validation matrix was plasma	[330]
TNF-α and IL-6	Inflammation and immune response	EC using chronoamperometry (CA)	rGO and AuNPs	LR = 0.1–500 pg/mL LOD = 0.69 pg/mL Recovery = NR	Antibody-based detection. Real samples were not tested.	[329]
IL-6	Colorectal cancer	EC using EIS	AuNPs	LR = 5 pg/mL–10 ng/mL LOD = 1.6 pg/mL Recovery = 103.9–109.6%	Aptamer-based detection. Real sample validation matrix was serum.	[331]
TNF-α	Inflammatory and autoimmune diseases	Fluorescence	ZnO NRs	LR = 0.1–100 fg/mL LOD = NR Recovery = NR	Antibody-based detection. Real samples were not tested.	[318]
Vaspin	Obesity and T2D	Fluorescence	UCNPs	LR = 0.1–55 ng/mL LOD = 39 pg/mL Recovery = 96.9–101%	Aptamer pair-based detection on an LFD. Real sample validation matrix was serum.	[328]
IL-6	Infectious disease	Fluorescence	CDs and AuNPs	LR = 1.5–5.9 pg/mL LOD = 0.82 pg/mL Recovery = 95.7–102.9%	Aptamer-based detection. Real sample validation matrix was serum.	[332]
IL-6	Inflammatory diseases	Colourimetric	Pd@Pt NDs	LR = 0.05–2 pg/mL LOD = 0.04 pg/mL Recovery = 93.7–105.5%	Nanozyme-based detection using antibodies. Real sample validation matrix was serum.	[333]
Visfatin	Metabolic disorder	Colourimetric	PtNP-modified Ce-MOF	LR = 1–100 ng/mL LOD = 0.11 pg/mL Recovery = 96.7–98.6%	Nanozyme-based detection using aptamer. Real sample validation matrix was serum.	[327]
RBP-4	Obesity and T2D	Colourimetric	AuNPs	LR = 0–125 nM LOD = 90.76 nM Recovery = NR	Salt-induced NP aggregation mediated aptamer-based detection. Real sample validation was not performed.	[312]
IL-6	Inflammation	Colourimetric	AuNPs	LR = NR LOD = 1.95 µg/mL Recovery = NR	Salt-induced NP aggregation-based detection by using aptamer-pair. Real samples were not tested.	[314]
IL-6	Infectious disease	Colourimetric	AuNPs	LR = 1 fg/mL–100 pg/mL LOD = 1 fg/mL Recovery = NR	Antibody-mediated detection on paper-based analytical device. Real sample validation matrix was human blood and bronchial aspirate.	[334]
IL-6	Cardiovascular disorders	Colourimetric	Magnetic beads	LR = 1.6–100 ng/mL LOD = 0.4 ng/mL Recovery = 99–114%	Antibody-based microfluidic detection. Real sample validation matrix was saliva.	[335]
TNF-α and IL-6	Immuno-modulation therapy	SPR	AuNRs	LR = NR LOD = 69.15 pg/mL (TNF-α) 0.93 pg/mL (IL-6) Recovery = NR	Multiplex detection using nano-plasmonic ink and antibodies. Practical validation was performed in an immunomodulated macrophage sample.	[316]
RBP-4	Acute kidney injury	SPR	MNPs	LR = NR LOD = 84 pg/mL Recovery = 96.7–98.6%	Smartphone-assisted antibody-mediated detection. Real sample validation matrix was urine.	[320]
IL-6	Cancer, cardiovascular disorders, diabetes, rheumatoid arthritis	SERS	Au@Ag-Au core–shell	LR = 100 fg/mL– 1 ng/mL LOD = 12.4 fg/mL Recovery = 92.4–105.3%	Antibody-based detection. Real sample validation matrix was spiked serum saliva and clinical serum samples.	[324]
Adiponectin	GDM	SERS	AuNTs	LR = 0.1 ng/mL–1 µg/mL LOD = 30 fg/mL Recovery = 93.1–106.02%	Antibody-mediated detection. Real sample validation matrix was serum from pregnant women	[315]
RBP-4	Obesity and T2D	CL	AuNPs	LR = 0.001–2 ng/mL LOD = 1 pg/mL Recovery = NR	Aptamer-based detection. Practical application was validated using patient samples.	[313]

^1^ This table is not exhaustive and only compiles studies from the last five years, 2019–2024.

## Data Availability

No new data were generated in this study. All data used in this review are publicly available via the references provided in the text.

## Abbreviations

1,5-AHG	1,5-anhydroglucitol
2 h PG	2 h postprandial plasma glucose
ADA	American Diabetes Association
Ag/Pt NCs	Ag/Pt nanoclusters
AgNWs	Silver nanowires
AIE	Aggregation-induced emission
ALP	Alkaline phosphatase
AuNPs	Gold nanoparticles
AuNPs	Gold nanorods
AuNP-VS_4_ NS NR	AuNP-vanadium sulphide nanosheet self-assembled nanorod
AuNTs	Gold nanotriangle
BCCAs	Branched-chain amino acids
BMI	Body-mass-index
CA	Chronoamperometry
CAGR	Compound annual growth rate
CDs	Carbon quantum dots
CGM	Continuous glucose monitoring
CHA	Catalytic hairpin assembly
CP	Carbon paper
CRET	Chemiluminescence resonance energy transfer
CRP	C-reactive protein
CV	Cyclic voltammetry
DALY	Disability-adjusted life year
DKA	Diabetic ketoacidosis
DPV	Differential pulse voltammetry
EC	Electrochemical
ECL	Electrochemiluminescence
EIS	Electrochemical impedance spectroscopy
ELISA	Enzyme-linked immunosorbent assay
Fe_3_O_4_ NPs	Iron oxide nanoparticles
Fe-Ni NPs	Iron-nickel nanoparticles
FGF-21	Fibroblast growth factor-21
FPG	Fasting plasma glucose
FRET	Fluorescence resonance energy transfer
GADA	Glutamic acid decarboxylase antibodies
GDM	Gestational diabetes mellitus
GNPs	Gold nanoparticles
GO	Graphene oxide
GOx	Glucose oxidase
H_2_O_2_	Hydrogen peroxide
HbA1c	Glycated haemoglobin
HCR	Hybridisation chain reaction
HDL	High-density lipoprotein
HLA	Human leukocyte antigen
HRP	Horseradish peroxidase
IA-2A	Islet antigen-2 antibodies
IAA	Insulin autoantibodies
IDF	International Diabetes Federation
IL-6	Interleukin-6
iSDA	Isothermal strand displacement amplification
LCR	Ligase chain reaction
LDL	Low-density lipoprotein
LFD	Lateral flow device
LOD	Limit of detection
LSPR	Localised surface plasmon resonance
MB	Methylene blue
MCH	6-mercapto-1-hexanol
MCP-1	Monocyte chemoattractant protein-1
miRNA	microRNA
MNPs	Magnetic nanoparticles
MOFs	Metal–organic frameworks
MRE	Biomolecular recognition element
MW	Methylene white
NCQ	Nitrogen-doped carbon quantum dots
NGAL	Neutrophil gelatinase-associated lipocalin
NIH	National Institute of Health
NR	Not reported
OCV	Open circuit voltage
OGTT	Oral glucose tolerance test
Pd@Pt NDs	Pd@Pt core@shell nanodendrites
PET	Photoinduced electron transfer
POC	Point-of-care
PPY	Polypyrrole
PtNP-Ce-MOF	PtNP-modified Cerium MOF
QDs	Quantum dots
qRT-PCR	Quantitative reverse transcription polymerase chain reaction
RBP-4	Retinol binding protein-4
RCA	Rolling circle amplification
rGO	Reduced graphene oxide
RPG	Random plasma glucose
SDA	Strand displacement amplification
SERS	Surface-enhanced Raman scattering
SHBG	Sex hormone-binding globulin
Si QDs	Silicon quantum dots
SMBG	Self-monitoring blood glucose
SPCE	Screen-printed carbon electrode
SPE	Screen-printed electrode
SPR	Surface plasmon resonance
SWV	Square wave voltammetry
ssDNA	Single-stranded DNA
T1D	Type 1 diabetes
T2D	Type 2 diabetes
TCNQ	7,7,8,8-tetracyanoquinodimethane
TFO	Triplex-forming oligonucleotide
TiO_2_NPs	Titanium dioxide nanoparticles
TMB	3,3’,5,5’-tetramethylbenzidine
tMB	Triplex molecular beacon
TNF-α	Tumour necrosis factor-α
TTF	Tetrathiafulvalene
UCNPs	Upconverting nanoparticles
WHO	World Health Organization
ZnO NPs	Zinc oxide nanoparticles
ZnO NRs	Zinc oxide nanorods
ZnT8A	Zinc transporter 8 antibodies
